# Genetic modeling of degenerative diseases and mechanisms of neuronal regeneration in the zebrafish cerebellum

**DOI:** 10.1007/s00018-024-05538-z

**Published:** 2024-12-27

**Authors:** Kazuhiko Namikawa, Sol Pose-Méndez, Reinhard W. Köster

**Affiliations:** Cellular and Molecular Neurobiology, Technische Universität Braunschweig, 38106 Braunschweig, Germany

**Keywords:** Zebrafish, Cerebellum, Purkinje cell, Granule cell, Degeneration, Spinocerebellar ataxia, Regeneration

## Abstract

The cerebellum is a highly conserved brain compartment of vertebrates. Genetic diseases of the human cerebellum often lead to degeneration of the principal neuron, the Purkinje cell, resulting in locomotive deficits and socio-emotional impairments. Due to its relatively simple but highly conserved neuroanatomy and circuitry, these human diseases can be modeled well in vertebrates amenable for genetic manipulation. In the recent years, cerebellar research in zebrafish has contributed to understanding cerebellum development and function, since zebrafish larvae are not only molecularly tractable, but also accessible for high resolution in vivo imaging due to the transparency of the larvae and the ease of access to the zebrafish cerebellar cortex for microscopy approaches. Therefore, zebrafish is increasingly used for genetic modeling of human cerebellar neurodegenerative diseases and in particular of different types of Spinocerebellar Ataxias (SCAs). These models are well suited to address the underlying pathogenic mechanisms by means of in vivo cell biological studies. Furthermore, accompanying circuitry characterizations, physiological studies and behavioral analysis allow for unraveling molecular, structural and functional relationships. Moreover, unlike in mammals, zebrafish possess an astonishing ability to regenerate neuronal populations and their functional circuitry in the central nervous system including the cerebellum. Understanding the cellular and molecular processes of these regenerative processes could well serve to counteract acute and chronic loss of neurons in humans. Based on the high evolutionary conservation of the cerebellum these regeneration studies in zebrafish promise to open therapeutic avenues for counteracting cerebellar neuronal degeneration. The current review aims to provide an overview over currently existing genetic models of human cerebellar neurodegenerative diseases in zebrafish as well as neuroregeneration studies using the zebrafish cerebellum. Due to this solid foundation in cerebellar disease modeling and neuronal regeneration analysis, the zebrafish promises to become a popular model organism for both unraveling pathogenic mechanisms of human cerebellar diseases and providing entry points for therapeutic neuronal regeneration approaches.

**Supplementary Information:**

The online version contains supplementary material available at 10.1007/s00018-024-05538-z.

## Overview: zebrafish cerebellum for modeling cerebellar disorders and regeneration

The cerebellum is one of the central brain compartments, essential for controlling smoothly coordinated movements and postural control [1] as well as for motor learning to aquire skillful actions [2]. Degenerative changes, or functional impairments of cerebellar neurons therefore lead to discoordinated movement, so called ataxia, which is a major clinical sign commonly observed in patients affected by cerebellar diseases [3]. In addition, cumulative evidence revealed the impact of the cerebellum on cognitive and emotional functions [4, 5], and cerebellar circuitry dysfunctions are thought to be associated with neurodevelopmental disorders accompanied by socio-cognitive and emotional impairments such as autistic behaviors [6]. Once such pathogical changes emerge, cerebellar functions and associated behavioral tasks are progressively impaired and hardly restored due to a lack in restorative or regenerative capacity of cerebellar neurons in humans as is the case for damaged neurons in other parts of the mammalian central nervous system [7]. To elucidate the molecular mechanisms underlying cerebellar disease etiologies, and further to develop therapeutic treatments for the disease conditions, genetically engineered mice have been generated, providing in *vivo* models that successfully recapitulate neuropathological changes as well as disease-associated behavioral impairments including ataxic behavior [8].

In addition to mice, zebrafish has gained popularity in the past years as in vivo model organism in cerebellar research. Indeed, over the last decade, research in zebrafish has contributed to deciphering development and physiology of the cerebellum, revealing that the zebrafish cerebellum displays a high degree of similarity compared to its mammalian counterparts with regard to genetic developmental programs, cellular dynamics and cytoarchitecture, neural circuits, and controlling behaviors [9, 10].

The zebrafish cerebellum contains nearly all neuronal populations corresponding to those in mammals, forming an evolutionary highly conserved cerebellar circuitry such as neuronal input into Purkinje cells (PCs) from parallel fibres (axons of the granule cells (GCs)) and climbing fibres (axons of the inferior olivery neurons). These principal cerebellar neurons are assembled during zebrafish development to form the typical three-layered cerebellar histology (molecular layer, Purkinje cell (PC) layer, and glanular cell (GC) layer) that is also found in humans [9, 10]. The functional cerebellar circuitry with the fast maturation of electrophysiological proterties of neurons, is established around 6–7 days postfertilization (dpf) in zebrafish [10, 11], which allows us to utilize these larvae for analyzing the physiological role of each neuronal subpopulation in cerebellum-controlled behaviors.

**Fig. 1:**
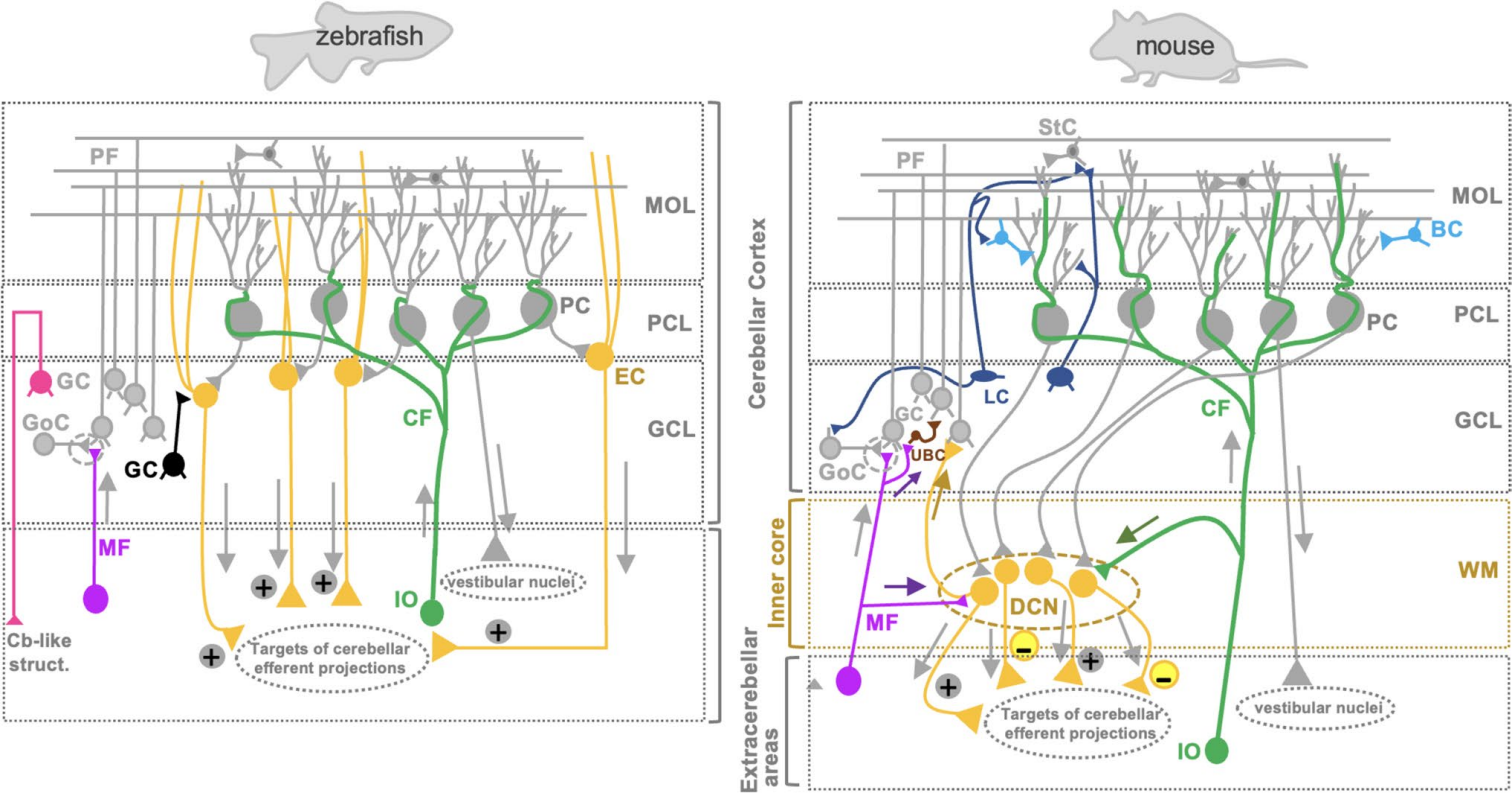
Schematic representation comparing cerebellar circuitry in zebrafish and mouse, showing conserved features (cells labeled in grey) and specific differences (differently colored cells). The main differences include: (I) additional projections from the mossy fibers to the deep cerebellar nuclei, and the unipolar brush cells in mouse (purple labeled), (II) additional projection from granule cells (GCs) to the cerebellar efferent cells or Eurydendroid cells (ECs) in zebrafish (black labeled), (III) climbing fibers reaching only proximal dendrites of Purkinje cells (PCs) in zebrafish vs. distal dendrites as well as additional projection to the deep cerebellar nuclei in mouse (green labeled), (IV) presence of basket cells (light blue labeled), unipolar brush cells (brown labeled), and Lugaro cells (dark blue labeled) only in mouse, (V) different distribution of efferent cells containing ECs dispersed between the Purkinje cell (PC) layer and granule cell (GC) layer in zebrafish, vs. neurons grouped in the deep cerebellar nuclei embedded in the inner core, which is composed of the white matter of the mouse cerebellum (orange labeled), (VI) presence of both excitatory and inhibitory efferent projections from deep cerebellar nuclei neurons in mouse (yellow labeled) vs. only excitatory projections from ECs in zebrafish that have been reported so far. (VII) Projections from axons of GCs to *crista cerebellaris* (magenta labeled) [17], a cerebellar-like structure present in zebrafish, but not in mouse [18]. The dotted circle indicates the cerebellar glomerulus, which consists of mossy fiber and Golgi cell terminals, along with granule cell dendrites, and is conserved in zebrafish and mouse. Abbreviations: Cb-like-struct, cerebellar like structures; CF, climbing fibers; DCN, deep cerebellar nuclei; EC, Eurydendroid cells; GC, granule cells; GCL, granule cell layer; GoC, Golgi cells; IO, inferior olive; LC, Lugaro cells; MF, mossy fibers; MOL, molecular layer; PC, Purkinje cells; PCL, Purkinje cell layer; PF, parallel fibers; StC, stellate cells; UBS, unipolar brush cells; WM, white matter. Details about the cerebellar neuroanatomy have been described recently in detail [10]

To investigate the roles of distinct neuronal subpopulations associated with the specific physiological processes of the cerebellum, zebrafish were genetically manipulated in a cell type-specific manner with genetically encoded calcium sensors, optogenetically controlled proteins, or those for conditional cell ablation. These approaches disclosed the importance of cerebellar neurons in regulating visuomotor behaviors such as optokinetic and optomotor responses [12–15], or in postural control with coordinated pectal fin-body movement especially during upwards swims [16]. These growing bodies of evidence suggest a great potential for expanding zebrafish cerebellum research to human disease modeling despite a few differences in cerebellar cytoarchitecture between zebrafish and mammals. For example, eurydendroid cells (ECs) that are functionally equivalent to efferent neurons in deep cerebellar nuclei in mammals, populate the PC layer or directly beneath it in the zebrafish cerebellum instead of forming condensed nuclei at the base of the cerebellum. In addition, a few types of mammalian cerebellar interneurons such as basket cells, Lugaro cells, and unipolar brush cells, have not been identified in zebrafish (Fig. 1) [9, 10].

Futhermore, a line of studies has revealed the tremendously high regenerative capacity of the zebrafish cerebellum to replace lost cerebellar neurons, an ability that is lacking in humans [19–22]. These findings have strongly attracting interest in deciphering this potent regenerative program in zebrafish. Thus, investigating the molecular mechanisms underlying cerebellar degeneration and regeneration in zebrafish enables one to gain insight not only into developing therapeutic interventions to protect neurons from neurodegeneration in humans, but also to replenish lost neurons by stimulating neuronal regeneration in the diseased cerebellum. The genetic tractability and transparency of zebrafish, especially during embryonic and larval stages, allow for in vivo imaging of disease-affected cerebellar neurons [23, 24]. In zebrafish, pathogenic genes can be coexpressed together with genetically encoded fluorescent reporters selectively in specific cerebellar cell types, their organelles and subcellular structures, enabling an in vivo cell biological analysis of disease-associated neuropathological changes during progression of cerebellar pathology [23–25]. The high fecundity of zebrafish, and the small size of larval zebrafish allow for the distribution of embryos and their maintenance in multi-well plates. This provides opportunities for testing and validating pharmaceutical compounds [26, 27] for therapeutic strategies, for example to stall disease progression, to reverse cerebellar pathology, or to further induce neuronal regeneration to re-establish a functional neuronal circuitry in the damaged cerebellum. In this review, we summarize cerebellar disease modeling in zebrafish, especially those for neuronal degenerative disorders, and update the knowledge about regenerative responses at the cellular and molecular level when neurons are invasively, or noninvasibly ablated in the zebrafish cerebellum.

## Modeling cerebellar neurodegenerative diseases in zebrafish

### Spinocerebellar ataxias (SCAs)

Degenerative Ataxia - also called Spinocerebellar Degeneration – represents a class of neurological diseases with progressive neurodegeneration primarily affecting neurons of the cerebellum. These diseases fall within the category of rare neurodegenerative diseases with an estimated prevalence of 15–20:100.000 [28], and encompasses genetic and sporadic types at a ratio of approximately 30–70%, respectively of all SCA cases according to a Japanese survey [29]. The most prominent clinical manifestation of degenerative ataxia is a compromised coordination of movements of the four limbs due to progressive cerebellar atrophy [3], most often caused by the loss of PCs, the most vulnerable neuronal population in the cerebellum [30, 31]. The patients further display additional major clinical signs attributed to cerebellar dysfunction, such as a poor coordination of eye movements and slurred speech [3]. Further neurological impairments including mental retardation and epilepsy accompany the main symptoms, which are specific for each SCA subtype. Most of the genetically caused degenerative ataxias are inherited in an autosomal manner. Autosomal dominant SCAs, typically named Spinocerebellar ataxias (SCAs) [32, 33], and autosomal ressesive SCAs, abbreviated SCARs [34, 35] appear with similar incidence rates (2.7:100,000 for SCA, and 3.3:100,000 for SCAR) [36]. The remaining small number of cases show X-linked dominant [37], or mitochondria-DNA inheritance [38].

### Genetic modeling of human neurodegenerative SCA diseases in zebrafish

Up to now, 39 genes that include 44 SCA loci, are registered in the Online Mendelian Inheritance in Men (OMIM) database [39]. These mutations are known to cause SCAs of which the encoding proteins fulfill diverse functions for example as transcriptional and translational regulators, ion-channels, kinases or phosphatases, protein homeostasis regulators or being involved in membrane trafficking [32, 33, 40]. Twelve out of these SCA-causing alleles carry expanded repeat sequences ranging from trinucleotide to pentanucleotide repeats found either in protein-coding or in non-coding sequences such as untranslated regions (UTR) or introns. Among them, CAG repeat expansions in protein coding sequences are translated to expanded polyglutamine (polyQ) stretches in the respective gene products, leading to the class of disorder known as polyQ disease which encompasses at least nine neurodegenerative diseases. These include Huntington’s disease, and spinal-bulbar muscular atrophy (SMBA), which selectively damage striatal neurons, and motoneurons, respectively, while the remaining seven polyQ diseases are referred to as polyQ-SCAs, characterized by cerebellar neurodegeneration among other pathologies [41]. PolyQ-SCAs includes SCA1, SCA2, SCA3/Machado-Joseph disease (MJD), SCA6, SCA7, SCA17, and Dentatorubral-pallidoluysian atrophy (DRPLA) [32, 33].

Neurodegenerative diseases are often accompanied by intracellular and/or extracellular deposits, such as Aβ42-plaques that accumulate in Alzheimer’s disese (AD) affected brains, Lewy-bodies associated with Parkinson’s disease (PD), and cytoplasmic aggregates involving TDP-43 in Amyotrophic Lateral Sclerosis (ALS) [42]. Similarly, nuclear and/or cytoplasmic inclusion bodies formed by polyQ proteins are a well-documented pathological hallmark manifested in degenerating neurons affected by polyQ diseases [30, 31]. As a specific feature of this class of disorder, polyQ diseases exhibit an inverse correlation between the age of onset and the number of expanded polyQ repeats, with a longer uninterrupted polyQ sequence resulting in an earlier age of onset and faster progression of disease symptoms [43]. Impariments in autophagy, a crucial system for maintaining neuronal protein quality, have been reported in animal models of cerebellar polyQ diseases, including SCA3/MJD, SCA6, SCA7, and DRPLA [44–47]. Among all neurons in the nervous system, cerebellar PCs are the most susceptible neuronal subpopulation affected by impaired autophagy processes, especially in juvenile mice [48, 49]. Thus, autophagy dysregulation impaired by polyQ proteins may affect PCs at younger stages, although the mechanisms underlying compromised autophagy differ among the different polyQ SCA diseases. Also, abundant expression of polyQ containing proteins in the cerebellum is considered critical for inducing cerebellar neurodegeneration pathologies, and at least for SCA6, the disease causing-gene is abundantly expressed in PCs [50]. These findings could explain the selective vulnerability of cerebellar neurons to polyQ-SCA proteins, as well as the broad temporal range of disease onset, which can initiate as early as childhood [51]. This contrasts with other neurodegenerative diseases that affect regions of the CNS other than the cerebellum, such as ALS showing a mid-to-late age onset, or late-onset degenerative disorders like AD and PD, where the initiation and progression of pathology are more closely associated with aging than in polyQ-SCAs.

On the other hand, expanded nucleotide repeats of CTG, ATTCT, CAG, TGGAA, GAA, GGCCTG, or ATTTC causing SCA8, SCA10, SCA12, SCA27B, SCA31, SCA36, SCA37, respectively, are found in non-coding sequence, and are thought to cause RNA toxicity [32, 33, 52]. Interestingly, the antisense strand of the CTG expansion sequence at the SCA8 locus encodes for CAG repeats, which are also transcribed in the opposite orientation by repeat-associated non-ATG translation [53].These repeats generate not only a polyQ protein product, but also polySer, and polyLys containing proteins. These poly amino acid stretches likely result in combinatorial toxicity together with the CTG expanded RNA [54]. The remaining SCAs such as SCA5, SCA13, or SCA28, are caused by alleles carrying missense mutations, insertions, or deletions [55–58], while genomic duplications were identidied in SCA20 and SCA39 loci [59, 60]. Moreover, ATX-THAP11 has been reported recently as a new polyQ disease caused by an extended polyQ stretch in the coding sequence of the *thap* gene [61].

**Fig. 2:**
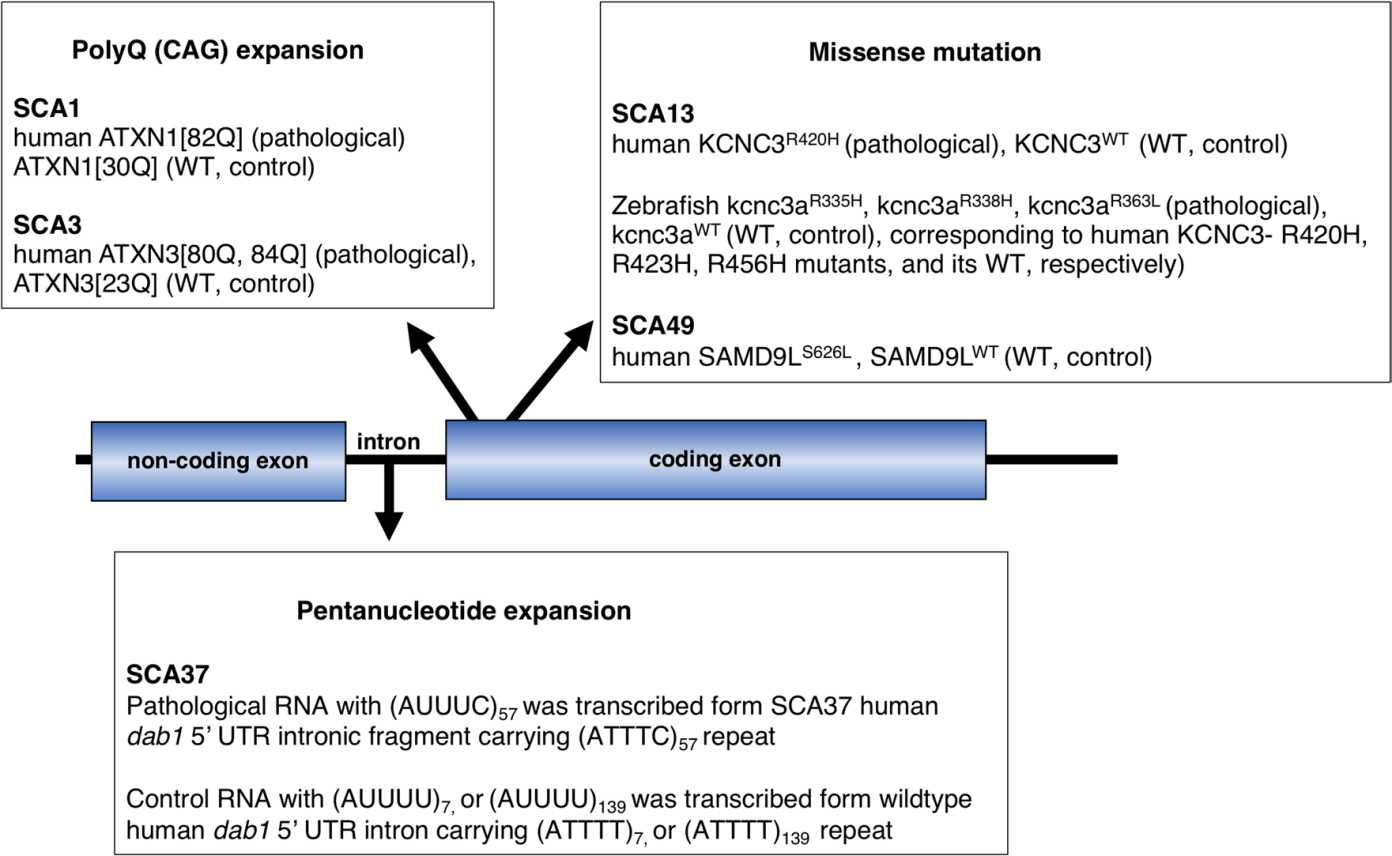
Schematic presentation of pathogenic transgenes to generate zebrafish models for respective SCAs. For simplicity, protein coding exons are depicted as a single box. Zebrafish modeling for SCA37 was achieved by injection of pathological RNA carrying (AUUUC)_57_, which was synthesized from the intronic fragment including the pentanucleotide (ATTTC)_57_ repeat in the 5′ untranslated region (UTR) of the *dab1* pathogenic allele, while RNAs containing (AUUUU) repeats from wild type alleles encoding (ATTTT)_7_ or (ATTTT)_139_ were used as controls [66]. The other zebrafish models for SCA1, 3, 13, 49 [23, 24, 67–73] were generated either by DNA, or mRNA injection to express protein coding sequences carrying CAG repeat expansions (for SCA1 and SCA3), or missense mutations (for SCA13 and SCA49), respectively. While most models were only generated as transient transgenic zebrafish, stable transgenic models were generated for SCA1 and SCA3 by raising DNA construct-injected embryos to adult zebrafish, screening the next generation for germline transmission and breeding the transgene through the following generations

The ideal animal models, representing the genetic conditions of human SCAs, are generated by replacing a wildtype allele with a disease causing allele with the help of targeted knock-in strategies, such as the Crispr/Cas9 technique [8]. This strategy allows for generating a toxic gain-of-function allele whose expression is spatio-temporally controlled by the endogenous regulatory elements and therefore such alleles mimic the human SCA causing gene expression pattern and strength more precisely. In addition, a knock-in approach simultaneously generates a haploinsufficiency (loss-of-function) of the wildtype allele, thus lowering the expression of the wildtype transcript and protein. However, such knock-in mutant mice sometimes displayed only a limited SCA pathology even during late stages of their lives due to the much shorter life span of mice compared to humans [62]. Indeed, artificially expanded CAG stretches in SCA mutant proteins, which were signficantly longer than stretches found in human patients, were needed to induce an apparent progressive SCA neuropathology in polyQ knock-in mice [63, 64]. Since the toxic gain-of-function SCA variants are thought to be the main cause of dominantly inherited SCA pathogenesis, mutant SCA allele overexpression in zebrafish - that can be readily established with the help of mRNA-injection or Tol2 transposon transgenesis [65] - have been reported for some of the SCAs so far (Fig. 2; Supplementary Table 1).

### Modeling of SCA3 in zebrafish

**Fig. 3:**
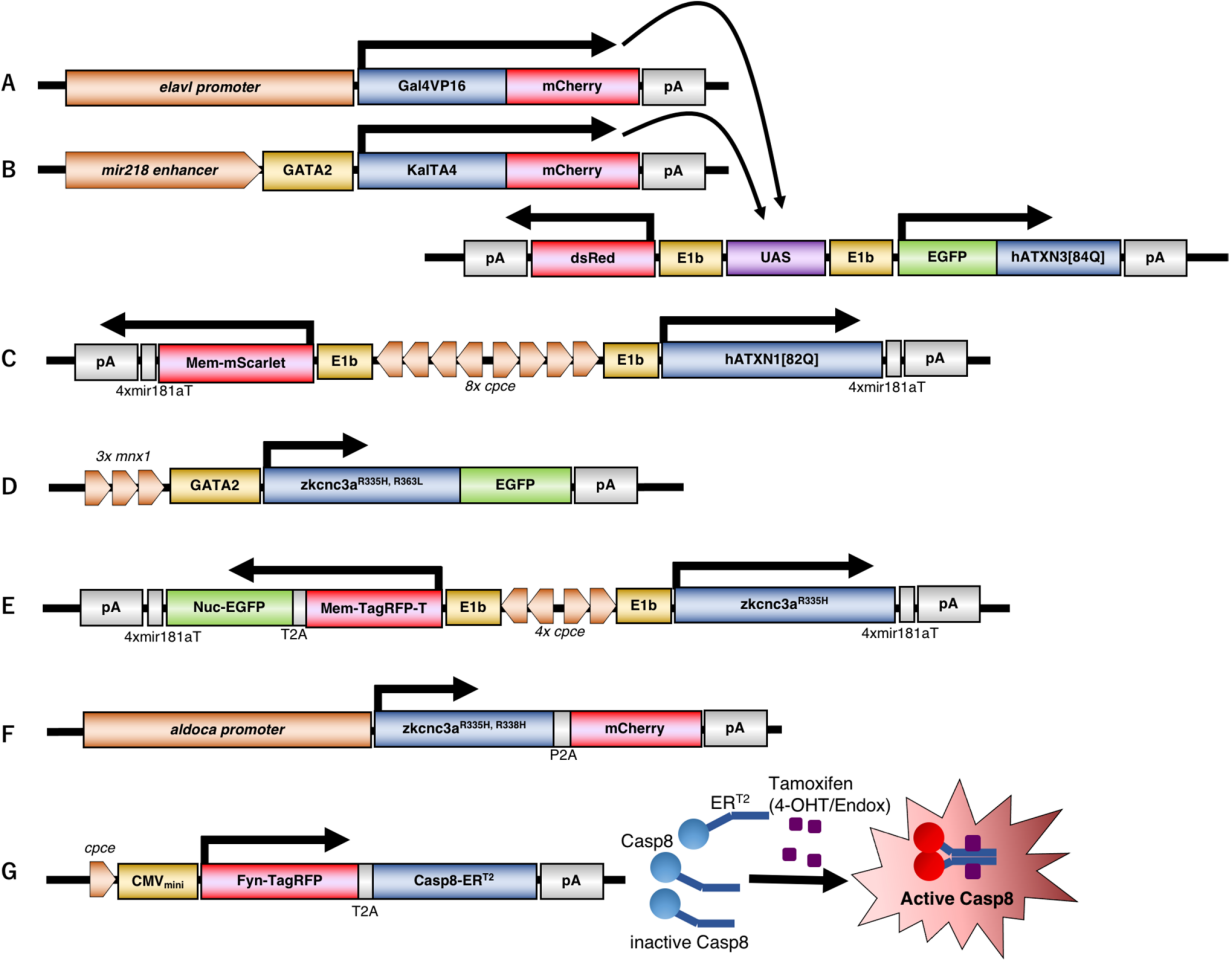
Schematic representation of cell type-specific expression cassettes for generating respective SCA (**A**–**E**) and acute cell ablation (**F**) models.** A** and **B** SCA3 zebrafish express Gal4VP16, or its variant KalTA4, in a pan-neuronal (**A**) or motoneuron specific (**B**) manner under the control of the *elavl3* promoter or mir218 enhancer fused to the *gata2* basal promoter. Translated Gal4VP16, or KalTA4 activates the expression of dsRed together with human SCA3[84Q] fused to EGFP from a bidirectional UAS responder unit [73]. **C** SCA1 zebrafish express humanATXN1[82Q] together with a membrane-targeted fluorescent protein mScarlet specifically in zebrafish cerebellar Purkinje cells (PCs) [23]. This transgene expression is mediated by a bidirectional expression construct using 8 copies in tandem of a PC specific regulatory element, *cpce*, flanked on both sides by E1b minimal promoters. To further restrict transgene expression to cerebellar PCs, a 4x miRNA181a target sequence (4xmiR181aT) was inserted between the coding sequence and the polyA (pA) sequence to eliminate ectopic transgene expression in tectal and hindbrain neurons. **D** The zebrafish kcnc3^R335H^ and kcnc3^R363H^ alleles, which are equivalent to the human SCA13 causing mutant alleles KCNC3^R420H^ and KCNC3^L456H,^ respectively, were fused to EGFP and expressed under the control of a motoneuron specific regulatory element composed of 3 tandem repeats of a *mnx1* (*hb9)* enhancer element (*3x mnx1*) linked to the *gata2* basal promoter [70]. **E** The same approach as for SCA1-modelling was used for generating a PC specific zebrafish SCA13 model, this time using 4 copies in tandem of the PC specific regulatory element *cpce*. A zebrafish kcnc3^R335H^ mutant   equivalent to the human SCA13 causing mutant allele KCNC3^R420H^, was coexpressed either with membrane-targeted mScarlet or a membrane targeted TagRFP-T (mem-TagRFP-T) together with nuclear localized localized EGFP (Nuc-EGFP) mediated by a self-cleaving T2A peptide [24]. **F** In an alternative approach, the zebrafish pathogenic mutant alleles zkcnc3a^R335H^ and zkcnc3a^R338H^, equivalent to human KCNC3^R420H^ and KCNC3^R423H^, respectively, were fused to P2A-mCherry to induce co-expression of each SCA13 mutant together with a red fluorescent reporter protein mCherry under the control of a PC specific *aldoca* promoter [68]. **G **A zebrafish PC-ATTAC line - allowing for drug inducible PC ablation - expresses the red fluorescent reporter protein TagRFP fused to T2A-Caspase8 (Casp8)-ER^T2^ (Tamoxifen-interacting estrogen receptor ligand-binding domain) [81]. This transgene allows for visualizing red fluorescent-labeled PCs, which can be ablated by the dimerization and activation of Casp8-ER^T2^, leading to apoptosis upon Tamoxifen treatment. 4-Hydroxytamoxifen (4-OHT), or Endoxifen (Endox), both active metabolites of Tamoxifen, were used for PC ablation in larval and adult zebrafish, respectively. The PC specific promoter is composed of the *cpce* regulatory element fused to a CMV minimal promoter

The first reported genetic model for human SCAs in zebrafish aimed at modelling the SCA3/MJD polyQ disease that is caused by an expanded CAG containing allele of *ATAXIN-3 (ATXN3)*, which encodes for a deubiquitination enzyme. Injection of mutant *ATXN3* mRNA containing a CAG_80_ repeat expansion encoding ATXN3[80Q] caused p53-dependent apoptosis that was mainly detected in the head of the zebrafish at 24hpf, possibly indicating neuronal degeneration [71]. To better illustrate a cell-autonomous neuropathology attributed to mutated ATXN3-polyQ expression in neurons at later larval and adult stages, a subsequent study described a stable transgenic line carrying an *ATXN3[84Q]* allele fused to EGFP that was expressed together with a cytoplasmic red fluorescent reporter protein [73]. Both transgenes were expressed under control of tandem repeats of upstream activating sequences (UAS), which serve as consensus binding site for the transcription factor Gal4VP16 (Gal4) [74] (Fig. 3A, B). When crossed with an *elavl3*:Gal4 zebrafish strain, expressing Gal4 in a pan-neuronal manner [75], double transgenic offspring carrying both Gal4 and UAS-transgenes induced ATXN3[84Q] expression together with the red fluorescent reporter in neurons throughout the entire nervous system (Fig. 3A) [73]. Compared to uninjected wildtype or ATXN3[23Q] expressing transgenic fish, such SCA3 zebrafish larvae displayed shortened spinal motor axons. With respect to behavior these SCA3 larvae swam shorter distances starting at 6dpf, a locomotor defect that lasted into adult stages. Furthermore, the average lifespan of these SCA3 zebrafish were reduced by 45 days compared to control fish expressing a wildtype ATXN3[23Q] allele [73]. When a Gal4 driver line *miR218*:Gal4 with a motoneuron specific miR218 enhancer [76] was used for breeding, ATXN3[84Q] expression in the offspring was restricted to motoneurons (Fig. 3B). These specimens exhibited a similar deficit in swimming as observed in zebrafish of the pan-neuronal SCA3 model (hereafter SCA3 model or SCA3 zebrafish) both at larval (6dpf) and adult (3 months) stages  [73]. Again, these motoneuron specific SCA3 zebrafish displayed shortened axons of spinal motoneurons, suggesting that the compromised movement observed in the SCA3 model is mainly caused by axonal dysfunction of motoneurons. Of note, one year old SCA3 zebrafish displayed accumulated polyQ protein as aggreates in neurites, especially in the medulla  [73], and such aggregates are a histological hallmark of polyQ-SCAs [30, 31, 77]. A subsequent study using flow cytometry revealed an increased number of fluorecent aggregates identified as detergent-insoluble ATXN3 particles in SCA3 larvae at 2dpf. These findings indicated an early initiation of polyQ protein related-pathology in SCA3 zebrafish even at early larval stages [78].

In addition, SCA3 zebrafish recapitulated the generation of toxic fragments of the ATXN3[84Q] protein generated by calpain-mediated proteolytic processing [73]. These proteolytic fragments are another pathological hallmark of SCA3 known from neurons derived from human SCA3 patients [79]. Of note, the impaired swimming of SCA3 zebrafish was restored when larvae were treated with the Calpain inhibitor Calpeptin, further supporting the impact of Calpain activation on SCA3-induced motor decificts. Noteworthy, SCA3 zebrafish treated with Calpeptin not only showed a decrease in ATXN3[84Q] cleavage fragments, but also a reduced level of full length ATXN3[84Q] protein expression [73]. More interestingly, Calpeptin treated SCA3 larvae displayed an enhanced autophagic activity [73], likely contributing to the removal of both full length and cleaved toxic fragment of ATXN3[84Q] in addition to the inhibition of Calpain-mediated ATXN3[84Q] proteolysis. Indeed, autophagy inhibition by active Calpain at multiple autophagy steps has been found in various pathological conditions [80], which also likely occur in zebrafish neurons affected by ATXN3[84Q]. Taken together, these results from the zebrafish SCA3 model strongly support the hypothesis that a toxic gain-of-function caused by the mutant ATXN3 protein, especially the appearance of toxic ATXN3 fragments generated by Calpain, is the central pathogenic event in SCA3-induced neuronal degeneration.

### Modeling of SCA1 in zebrafish

Despite such successful use of SCA3 zebrafish, broad expression of the ATXN3 mutant protein throughout the CNS or its restricted expression in motoneurons still raises the question of whether such models could recapitulate the brain region-specific pathology commonly observed in individuals with neurodegenerative diseases [82]. Indeed, approximately half of the SCA3 patients display peripheral neuropathy [83], which has been successfully replicated in the pan-neuronal and motoneuron specific SCA3 zebrafish. However, it remains largely unknown whether the SCA3 mutant protein damages specific brain regions in addition to the spinal motoneurons in zebrafish and whether these affected neuronal populations in the brain contribute to zebrafish behavioral impairment corresponding to human ataxia.

Specific distribution patterns of disease-associated proteins are considered to be strongly associated with brain region- and/or neuronal population-specific damage, leading to individual disease symptoms. Thus, for genetic disease modeling it is preferable to target vulnerable neurons specifically affected in the respective disease to be studied using neuronal cell type specific regulatory elements. Since cerebellar PCs are regarded as a neuronal population primarily affected in SCA diseases [30, 31], PCs have been targeted when SCA transgenic mice models were generated, which successfully displayed ataxic-like behavior [84]. We therefore sought to develop SCA zebrafish coexpressing a SCA causative mutant together with fluorescent reporters in PCs, allowing for monitoring the progression of SCA affected neurons [24]. Towards this, we isolated a compact PC-specific regulatory element designated *carbonic anhydrase 8 promoter-derived PC specific enhancer element* (*cpce*), which could be multimerized for increasing expressing strength in PCs in a step-wise manner. This series of enhancers with different strengths were used to establish bidirectional bicistronic PC-specific co-expression constructs. Varying copy numbers of the *cpce* element allows one to adjust the dose of a SCA causative gene product expressed in PCs, thereby inducing fast, moderate, or slow progression of the PC pathology dependent on the respective SCA mutant. With help of this PC-specific co-expression system, we generated two zebrafish models for SCA1 and SCA13 respectively, which are either caused by a polyQ expansion (SCA1) or a missense mutation in the disease-causing protein (SCA13) [23, 24].

The SCA1 causative allele encodes for a nuclear protein ATAXIN-1 (ATXN1), which binds to transcription and splice factors. The dysregulation of these factors by the ATXN1-polyQ mutant is proposed to be crucial for SCA1 pathogenesis [85]. Zebrafish contain two homologous genes to human *ATXN1*, and their transcripts were detected throughout the nervous system including PCs [86], which prompted us to conduct genetic modeling of SCA1 in zebrafish. We generated a stable transgenic SCA1 zebrafish coexpressing human ATXN1[82Q], or its wild type form ATXN1[30Q] as control, with a red fluorescent reporter protein under the control of *8xcpce* with strong PC enhancer activity (Fig. 3C). Neurodegenerative changes of PCs in SCA1 transgenic fish successfully initiated at around 6 weeks post-fertilization (6wpf) of the late larval stage, and progressed continuously throughout the juvenile stages to the young adult stage of 3 months. At this age most of the red fluorescent-labeled PCs were almost entirely eliminated except for a few remaining PCs in the caudal region of the cerebellum [23]. In comparison, the control line expressing wild-type ATXN1[30Q] in PCs did not show any sign of neurodegeneration. SCA1 fish, displaying progressive PC degeneration at 2 and 3 months of age, resulted in reduced exploratory behavior, while an obvious deficit in locomotive performance could not be observed. These results suggest that zebrafish PCs play an important role in controlling socio-emotional activity with a less obvious impact on locomotion, unlike PCs in mammals, which crucially regulate both behaviors. Nevertheless, SCA1 zebrafish will facilitate investigations about the causative cell biological processes underlying polyQ-induced PC degeneration, because these fish allow for in vivo imaging especially at larval stages to understand the molecular mechanisms underlying initiation of SCA1 pathology. Furthermore, these zebrafish larvae and juveniles will be useful for pharmalogical compound testing to identify SCA1 inihibitors or compounds that exert neuroprotective functions.

### Modeling of SCA13 in zebrafish

The onset of SCA13 can either occur already during infancy or displays first symptoms during adulthood with neurological symptoms encompassing motor discoordination, dysarthria, mental retardation, and epilepsy [87]. SCA13 is caused by alleles containing missense mutations or a deletion in a voltage-gated potassium channel, KCNC3/Kv3.3. This channel enables cerebellar PCs to fire repetitively at high frequencies [56, 87]. The zebrafish genome carries two genes homologous to human KCNC3, named *kcnc3a* and *kcnc3b* [88], and one of them, *kcnc3a*, is prominently expressed in cerebellar PCs in zebrafish [24]. The Kcnc3a and Kcnc3b proteins show highly conserved channel functions compared to human KCNC3. Amino acid substitutions mimicking human mutations causing SCA13 in the respective homologous proteins in zebrafish result in impaired electrophysiological properties as observed in their human SCA13-causing homologous counterparts [88]. So far, modeling human SCA13 in zebrafish was achieved by transiently overexpressing the human adult-onset mutated allele KCNC3^R420H^ [69] and its zebrafish corresponding mutant (*kcnc3a*^*R335H*^) [24, 68, 70]. Similarly, two zebrafish variants (*kcnc3a*^*R338H*,^ and *kcnc3a*^*R363L*^) were used in transient transgenic models, which are equivalent to infant-onset SCA13 mutants of KCNC3^R423H^ and KCNC3^L456H^ [68, 70], respectively. Previous studies based on ubiquitous expression of human adult-onset KCNC3^R420H^ [69] or motoneuron specific expression of its zebrafish homolog *kcnc3a*^*R335H*^ as well as the infant-onset *kcnc3a*^*R363L*^ (Fig. 3D) failed to elicit clear neuronal degeneration  [70]. Yet, caudal primary motoneurons overexpressing either *kcnc3a*^*R363L*^ or *kcnc3a*^*R335H*^ showed developmental defects such as axonal pathfinding errors, or compromised arborization of distal axonal arbors [70]. These results empasize the importance of modeling SCA13 by specifically targeting cerebellar PCs as the primary SCA13-affected neuronal population.

We therefore exploited our tunable PC-specific expression system to generate transient transgenic SCA13 larvae coexpressing *kcnc3a*^*R335H*^ together with fluorescent reporters in zebrafish PCs (Fig. 3E). This PC-specific SCA13 model successfully induced fragmented axonal and/or dendritic structures of PCs, starting at 5-6dpf, and such neurodegenerative changes progressed until 2 weeks postfertilization (2wpf). SCA13 larval fish further displayed an impaired optokinetic response with irregular eye pursuit movements and fewer numbers of saccades [24], which reminds of eye movement deficits manifested in SCA patients [89]. Using zebrafish *kcnc3a*^*R338H*^ the mutant allele equivalent to infant-onset human SCA13^R423H^ as well as *kcnc3a*^*R335H*^, Hsieh et al., further addressed electrophysiological alterations and morphological consequences of these mutant alleles when expressed in zebrafish PCs (Fig. 3F)  [68]. *kcnc3a*^*R338H*^ caused dramatic and transient hyperexcitability of PCs with affected PCs being impaired in neurite extension as well as dendrite- and synapse formation, leading eventually to rapid neuronal death within 7-8dpf. In contrast, PCs expressing *kcnc3a*^*R335H*^ displayed reduced excitability during evoked high-frequency spiking, and these PCs developed and matured normally without any sign of degeneration [68].

These findings are inconsistent with our observation of clear PC degeneration induced by *kcnc3a*^*R335H*^ overexpression [24]. This discrepancy is likely due to the difference in the strength of PC-specific promoters used in both models. Although both models used transient transgenic specimens expressing *kcnc3a*^*R335H*^, Hsieh et al. used the upstream region of the *aldoca* gene whose PC-specific promoter activity is much weaker than those driven by 4x*cpce*, and 2x*cpce* (our unpublished observation) both of which induce clearly progressive PC degeneration in the larval cerebellum [24]. The *cpce*-enhancer mediated models allow for making use of the power of zebrafish, such as in vivo imaging, and validation of compounds using multi-well plate assays. However, caution is still needed in the interpretation of outcomes from the adult-onset mutant-induced neuronal degeneration when larval zebrafish are used. Both, *cpce* enhancer and *aldoca* promoter drive pathological gene expression starting during differentiation stages of postmitotic PCs. As a result, SCA13 mutants impact on developmental processes in PCs, including dendritic and axonal growth, as well as the establishment of cerebellar circuitry. To further refine models for PC degeneration, that more accurately reflect the adult-onset SCAs, an inducible gene expression system, such as CreER-loxP, can be employed in combination with the *cpce* enhancer to selectively target fully matured PCs. These cells, having reached both anatomical and functional integration into cerebellar circuits, can be specifically manipulated in zebrafish at later larval, juvenile or adult stages. Although the current models still face such limitations, zebrafish disease modeling of human SCA13 will allow for in vivo imaging, electrophysiological studies, as well as behavioral analysis, and therefore offer a comprehensive analysis to understand molecular pathogenesis underlying both infant and adult-onset SCA13.

### Other SCA and related models in zebrafish

#### SCA37

In addition to above-memtioned SCA models, the following transient transgenic zebrafish were generated by microinjection of mRNA encoding respective SCA mutants. As a causative allele for SCA37 dysplaying pure cerebellar ataxia with PC loss, Seixas et al. identified the pathogenic pentanucleotide ATTTC repeat expansion in the 5′UTR intron of the *Disabled 1 (DAB1)* gene [66]. DAB1 is an intracellular adaptor protein involved in Reelin signaling, regulating the migration and positioning of neurons to establish proper brain laminar structures including those in the cerebellum [90]. Contrary to the (ATTTT)_7−400_ repeats in the *Dab1* wild type allele, the SCA37 mutated allele contains the pathogenic repeat expansion (ATTTC)_31−75_ flanked by (ATTTT)_60−90_ repeats. The RNA encoding the *DAB1* intronic fragment carrying pathogenic repeats (AUUUC)_57_ formed nuclear foci, when expressed in cultured cells, whereas those of wild type alleles such as (AUUUU)_7_, or (AUUUU)_139,_ showed no pathological phenotype [66], indicating RNA toxicity of the SCA37 pathogenic intron. The in vivo toxicity was next examined by microinjection of these RNAs into zebrafish embryos, revealing lethal developmental malformations caused by the expression of the (AUUUC)_57_ insertion RNA, but not by the wild type (AUUUU) repeat RNAs [66]. investigations using postmortem SCA37 affected cerebellar tissue has shown robust PC loss, whilst remained PCs display perisomatic and perinuclear punctate staining of DAB1 [91]. Furthermore, dysregulated DAB1 expression was observed in these spiecemens, for example, the generation of *DAB1* pathogenic transcripts and significantly higher levels of DAB1 protein [91]. Impact of DAB1 function on cerebellar lamination in zebrafish has been provided from studies on homozygous mutants of *dab1a* (one of the zebrafish *dab* alleles homologous to human *DAB1*), resulting in mispositioning of ectopically produced cerebellar cells including PCs at adult stages [92]. In addition to DAB1 dysregulation, an aberrantly activated PI3K/Akt pathway is found in cerebellar tissue from SCA37 patients [91]. Interestingly, this cascade has been previously identified as an enhancer of disease progression in a SCA1 *Drosophila* model [93]. Thus, a future approach should target zebrafish PCs expressing the *DAB1* pathogenic intron to study dysregulated DAB1 and PI3K/Akt signaling in these neurons, in which degeneration might be induced.

#### SCA49

SCA49 is caused by a missense mutation in the *SAMD9L* gene [67] whose heterologous gain-of-function alleles have been described previously in causing the Ataxia-pancytopenia syndrome. This disease is characterized by neurological symptoms including cerebellar ataxia as well as pancytopenia frequently associated with hypocellular bone marrow protein [94]. For example, SCA49 patients carrying the *SAMD9L*^*S626L*^ missense allele predominantly show neurological defects of cerebellar origin, such as ataxia, eye movement disturbances, and dysarthria with additional sensory axonal polyneuropathy [67]. Although the SAMD9L protein is proposed to have anti-proliferative functions [95] and to regulate endosome fusion [94], its neuronal function is poorly investigated. Larval zebrafish injected with mRNA encoding human wild type SAMD9L, or its S626L mutated allele causing SCA49, revealed a localization of the respective proteins in mitochondria in neurons of the hindbrain and the spinal cord. Zebrafish injected with *SAMD9L*^*S626L*^ mRNA showed no obvious morphological changes or increased lethality. Yet, their protein lysates contained increased levels of the mitochondrial fusion protein DRP1 and mtDNA, suggesting enhanced mitochondria biogenesis, which was interpreted as a compensatory response to mitochondria stress [67]. Indeed, these observations were also recapitulated in human SCA49 patient-derived fibroblasts, confirming the siutability of zebrafish in vivo models for SCA analysis. With respect to behavioral alterations, larval fish with ectopic expression of wild type SAMD9L displayed more motile swimming with increased number of head turns during light-dark cycles compared to control zebrafish, indicating enhanced neuronal activity mediated by wild type SAMD9L in the motor and vestibular-sensory system. Larval zebrafish expressing the mutant S626L instead, failed to show behavioral hypo- or hyperperformance compared to controls, although peripheral neuropathies are manifested in SCA49 patients. Thus, this zebrafish model still leaves open questions about SCA49 mutant-induced toxic gain-of-functions, potentially mediating neuronal degeneration and behavioral alterations. Currently, only the enhanced mitochondria biogenesis appears to be conserved between this zebrafish model and SCA49 affected human cells [67].

Although these two studies describing SCA37 and SCA49 zebrafish demonstrated the feasibility of rapid cytotoxicity testing of newly identified SCA mutants in zebrafish, further models mediating SCA causing gene expression in cerebellar neurons, especially in PCs will still be needed. Such models will allow for verification of mutant allele toxicities and intracellular changes in PCs, while analysis of behavioral consequences relevant to impaired cerebellar functions will provide structure-function relations for pathogenic SCA allels. Cell type-specific pathogenic gene expression also enables one to avoid embryonic lethality caused by RNA injections that induce ubiquitous expression of toxic genes, as observed in the case of the SCA37 zebrafish model.

#### SCA2 and amyotrophic lateral sclerosis (ALS)

SCA2 belongs to the polyQ SCAs. It is caused by a CAG expanded (≥ 34) variant of *ATAXIN-2* (*ATXN2*) whose gene product is involved in regulating RNA metabolism [96]. Although the toxicity of a SCA2 pathogenic variant with a long expanded CAG repeat has not been tested in zebrafish yet, its variant *ATXN2[30Q]* carrying the intermediate CAG length of 30Q was examined in zebrafish especially in the paradigm of amyotrophic lateral sclerosis and frontotemporal dementia (ALS-FTD) [97]. This is important, because ATXN2 with the intermediate polyQ length of Q27-33 is the most important risk factor for ALS-FTD [98]. Overexpression of RNA for *ATXN2[30Q]*, or morpholino-induced partial reduction (50%) of the expression of C9orf72, one of the ALS-FTD causative genes, failed to show any phenotypical alteration of motoneurons and behaviors. In contrast, pathological changes in motor axons as well as reduced touch-evoked startle behavior were observed in larvae coinjected with a c9orf72 morpholino together with mRNA encoding *ATXN2[30Q]*, but not *ATXN2[22Q]* [97]. Thus, this study confirmed ATXN2 with the intermediate polyQ length as a genetic enhancer of ALS-FTD in zebrafish, but SCA2 pathogenic variants with longer polyQ (≥ 34) still need to be examined in the context of SCA2 with respect to their toxic effects especially affecting cerebellar neurons in zebrafish.

#### Other SCAs–loss of function effects

Although, SCA pathogenic alleles are considered to exert a toxic gain-of -function as dominant driver of cerebellar degeneration, additive detrimental effects of wildtype protein haploinsufficiency, enhancing a toxic gain-of-function of SCA mutants, are also likely, which was reported at least for a SCA1 mouse model [99]. It is therefore interesting to examine the loss-of-function phenotypes of wildtype alleles whose pathogenic variants cause SCAs. Hence, zebrafish morphants and crispants, or mutants identified from ENU mutagenesis projects were used to investigate the consequences resulting from the diminished expression of their wildtype alleles linked to SCA3, SCA6, SCA7, SCA14, or SCA17 [100–107] (Supplementary Table 2). Out of these, pathological concequences for the cerebellum were observed in morphants for *ataxin7 (atxn7)* whose human allele *(ATXN7)* encoding an expanded polyQ sequence is causative for SCA7. *ATXN7* encodes a subunit of the Spt-Ada-Gcn5-acetyltransferase (SAGA) complex acting as a chromatin modifying factor [108]. Moderate morpholino-mediated downregulation of *atxn7* expression in zebrafish inhibited the differentiation of retinal photoreceptors, cerebellar PCs and granule neurons [104]. Indeed, cone-rod retinal dystrophy is a main pathological mainfestication in SCA7 patients in addition to cerebellar degeneration [109]. A further study using both *atxn7* morphants and crispants confirmed impaired eye development caused by the decrease of *atxn7* expression. The observed phenotypes included not only a compromised terminal differentiation of photoreceptors, but also eye patterning defects resulting in ocular coloboma, which are associated with reduced expression of a photoreceptor specific transcription factor, Crx, and increased Shh signaling, respectively [105]. Thus, these studies revealed essential roles of *atxn7* during cerebellar differentiation and eye development, especially photoreceptor terminal deffirentiation. The findings also suggest that a partial loss-of-function of *ATXN7* contributes to SCA7 pathogenesis. The generation of transgenic lines expressing SCA mutated alleles for SCA7 as well as other SCAs will be needed to examine their toxic gain-of-functions.

### Therapeutic compound testing using a genetic zebrafish model for SCA3

Genetic modeling of neurodegenerative diseases in zebrafish provides an opportunity to test and validate compounds that slow down disease progression, or even revert neuropathological changes of the disease-affected neurons. The candidate compounds can be easily added in a dilution series to the rearing medium of zebrafish larve. These can be maintained in multi-well plates to easily identify the optimal therapeutic concentration without deleterious side effect. Furthermore, a functional blood brain barrior is established in zebrafish larvae by 5dpf [110], allowing to verify if a compound of interest is able to pass into the brain parenchyma. A validation of compound activity could be based on the morphological restoration of a disease-affected cerebellum, since it is accessible for in vivo imaging. Alternatively, the behavioral recovery of zebrafish exposed to candidate compounds could be monitored, and both assays can be combined. The potential of zebrafish model for testing therapeutic compounds against SCAs has been confirmed using SCA3 zebrafish described above, which were used to validate BLD-2736 as a novel calpain inhibitor [72]. BLD-2736 worked effectively at an approximately 10 times lower dose compared to Calpeptin in removing fragmented and aggregated ATXN3-polyQ, likely by Calpain inhibition and activation of autophagy in SCA3 zebrafish larvae, and further promoting their behavioral recovery. This fish model was also used to examine the therapeutic potential of the HDAC inhibitor sodium valproate [111] known as a protective compound against SCA3 toxicity [112, 113]. This study revealed the high efficacy of sodium valproate in restoring impared swimming in SCA3 larvae [111]. Subsequent proteomic analysis using protein lysates from valproate treated SCA3 larvae identified the Sirtuin longevity pathway as a downstream signal activated by valproate [111]. Resveratrol induced pharmacological activation of Sirt1, a key nuclear deacetylase involved in the Sirtuin pathway [114], alleviated behavioral deficits in SCA3 fish and enhanced autophagy. Indeed, Sirtuin activation is well-known to activate autophagy [115]. In contrast, SCA3 larvae cotreated with valproate and EX527, an inhibitor of Sirt1, resulted in reduced autophagy and a decreased swim activity, that was enhanced by the treatment with valproate alone. These findings further support the importance of the Sirtuin-autophagy pathway contributing to the rescue of valproate-treated SCA3 zebrafish.

These results further underscore the impact of autophagy stimulating compounds in protecting neurons from SCA3-induced neuronal degeneration [111]. In a subsequent study, SCA3 larvae were treated with four autophagy activating compounds: loperamide, trehalose, rapamycin, and MG132, each of which ameliorated motor impairment of SCA3 larvae [116], demonstrating the feasibility of in vivo screening of autophagy activators in SCA3 zebrafish [117]. In addition, sodium butylate, another HDAC inhibitor, which mitigates ataxic behavior in SCA3 transgenic mice [118], was examined on SCA3 zebrafish larvae, resulting in elevated autophagy and improved swimming performance [119]. This increase in autophagy is thought to occur at the level of autophagy initiation, because AMPK protein and phosphorylation of its substrate ULK1 were increased in protein lysate extracted from SCA3 larvae treated with sodium butylate [119]. Collectively, these results underscore the impact of autophagy-stimulating compounds in protecting neurons from SCA3-induced neuronal degeneration.

### Therapeutic compound testing using genetic zebrafish models for other cerebellar neurodegenerative diseases

Studies using SCA zebrafish models for compound testing are still limited to SCA3 fish, and it still remains elusive whether compounds effectively alleviating SCA3 neuropathology equally revert those in SCA1, or SCA13 zebrafish. However, the increasing number of zebrafish models for cerebellar neurodegenerative diseases other than SCAs provides the opportunity to validate the effectiveness of therapeutic compounds on suppressing cerebellar pathology in general or their limited use for a specific disease condition.

Autosomal recessive spastic ataxia of Charlevoix-Saguenay (ARSACS) is a complex neurodegenerative disorder, which represents early children onset spastic ataxia and peripheral neuropathy as main clinical hallmarks. Over 400 ARSACS causing mutations were found in a SACS gene encoding the Sacsin protein, which is though to modulate the ubiquitin-proteasome system and the Hsp70 chaperone machinery [120]. Mutant mice with a non-functional null allele of the *Sacs* gene show progressive PC loss and motor impairment, which revealed that the loss of function of SACS is toxic [121]. Crispr/Cas9-mediated *sacs* null mutant zebrafish were therefore generated, successfully resulting in reduced cerebellar tissue with enhanced Ca^2+^-dynamics in the remaining PCs, further displaying impaired free-swimming at early larval stages of affected zebrafish [122]. In contrast, morphological deficits of sensory and motor neurons, or muscles were absent in these mutants, despite the symptoms of pheripheal neuropathies and muscle spasticity in ARSACS patients. *sacs* mutant zebrafish were treated with two FDA-approved compounds: Acetyl-DL-leucine, a stimulator of intracellular glutamate metabolism, and an ER stress inhibitor TUDCA, both of which are being clinically tested for other types of hereditary ataxia than ARSACS [123, 124]. Although effects of these compounds in suppressing the ARSACS PC pathology were not described in this study, both treatments successfully improved swimming in *sacs*^−/−^ larvae [122], indicating at least a functional recovery of the compromised cerebellum or other brain regions associated with locomotive behavior.

Testing of pharmaceutical compounds was also examined on a zebrafish model displaying neurodevelopmental disorders accompanied by cerebellar disorganization. We generated a transgenic zebrafish strain– PC-Dyrk1A - co-expressing the green fluorescent protein mClover as reporter together with human Dual-specificity tyrosine phosphorylation–regulated kinase 1 A (Dyrk1A) specifically in PCs, which endogenously express zebrafish homologues of this kinase [125]. Dyrk1A is a protein kinase phosphorylating serine/threonine residues of a broad range of proteins that have crucial roles in neurons. Both Dyrk1A increased expression or its reduced expression are cumulatively reported to be associated with neuropathologies in various neurological diseases such as Down Syndrome (DS), Alzheimer’s Disease (AD), Huntington’s Disease (HD), and autistic spectrum disorder (ASD) [126–129]. Especially, the neuropathogenic role of Dyrk1A hyperactivation has been well-described in DS [130], which is caused by trisomy of human chromosome 21 (HAS21) containing the Dyrk1A gene [131]. Overdose of Dyrk1A by 1.5-fold changes in DS is thought to be one of the main drivers to induce neurodevelopmental deficits including mental retardation as a typical DS symptom, and early onset cognitive impairment that is also a frequent manifestation in DS individuals [130]. The prominently smaller mass of DS brains including the cerebellum is obvious compared to those in healthy individuals [132], which is recapitulated in DS mice models such as the Ts65Dn mice carrying the triplicated chromosomal region containing the Dyrk1A gene [133]. Normalization of Dyrk1a dosage by genetic deletion of a single copy of the Dyrk1A gene from DS mice can partially rescue their cerebellar pathologies, displaying an increased density of GCs and PCs, and normalized dendritic arborization of PCs, further supporting the idea that Dyrk1A contributes to neuropathologies of cerebellar neurons in DS individuals [134].

In vivo imaging of the genetic zebrafish PC-Dyrk1A model revealed no pronounced degeneration of PCs at larval stages at 4-7dpf, but condensed cerebellar hemispheres with more densely organized PCs likely caused by less complex PC dendrites compared to controls [125]. This disorganization of the PC layer progressed until adulthood at which PCs with Dyrk1A hyperactivity failed to maintain a characteristic layered structure of the typical adult cerebellar PC population. Instead, these neurons rather distributed deeply into the molecular layer with significantly reduced synaptic contacts inside it, indicating that Dyrk1A-affected PCs are not properly integrated into cerebellar circuits. These fish displayed a reduced exploratory behavior, consistent with the behavior of SCA1 fish with pronounced PC degeneration in which PC function is lost [125]. In contrast, reduced *dyrk1a* expression resulted in opposite phenotypes such as increased exploratory and anxiolytic behavior [135]. Interestingly, already during larval stages at 6dpf, PC-Dyrk1A zebrafish showed a reduced activity in swimming, indicating that their PC disorganization starts to affect behavior even at early larval stages [125].

These larvae provided a good testing ground for Dyrk1A-inhibitors targeting its kinase activity. A number of Dyrk1a kinase inhibitors have been identified and validated in cultured cells or Dyrk1A overexpressing animal models such as *Drosophila* or mice [136, 137]. However, the most selective inhibitor, KuFal194, had not been tested in vivo so far [138]. When added to the rearing medium of zebrafish PC-Dyrk1A larvae, this inhibitor was able to revert the disorganization of the larval PC hemispheres similar to previously established inhibitors such as ProINDY and Leucettine L41 without showing any detrimental side effects [125]. These results reinforce that genetic modeling of human cerebellar diseases using zebrafish is suitable for compound testing, and shed light on KuFal194 as a promising candidate for further validation towards its therapeutic use against DS pathology.

Furthermore, there are currently growing numbers of reports describing genetically modified zebrafish for cerebellar diseases inherited in an autosomal ressesive manner, which include models for SCARs and other neurodevelopmental disorders displaying cerebellar hypoplasia and/or malformation (Supplementary Table 3). The appearance of these cerebellar pathologies caused by loss-of-function of respective disease-causing genes have been examined in zebrafish injected with morpholinos [139–149], with CRISPR/Cas9-mediated gene depletion [122, 150], or those identified from ENU-induced mutation screening projects [145, 151]. Since these zebrafish successfully mimicked respective disease phenotypes in the cerebellum, they will be available for testing compounds whose efficiency in rescuing cellular phenotypes can be easily validated based on the morphological recovery of cerebellar structures with the help of imaging analysis.

### Genetic models of acute neurodegeneration of cerebellar Purkinje cells

Genetic models for human neurodegenerative diseases of the cerebellum established in zebrafish are valuable not only to investigate the molecular pathology of SCA-affected neurons in vivo, but also to examine the consequences of PC loss with respect to physiological functions of the cerebellum. However, neurodegeneration in cerebellar disease models progresses gradually dependent on the respective model, for example within 1–2 weeks in SCA13 [24], or 6 weeks-3 months in SCA1 zebrafish [23]. Furthermore, a regional difference in the vulnerability of PCs within the PC population was observed at least in the SCA1 model [23]. These observations cannot rule out a possible compensatory role of rewired cerebellar connections by remaining PCs in the SCA-affected cerebellum. Thus, an alternative model is needed to interpret the physiological significance of PC function in the context of cerebellar circuits, which is further relevant to analyze cerebellum-controlled behaviors.

Ideally, such a genetic model achieves a near to complete and acute PC ablation, allowing one to clearly reveal the physiological and functional consequences caused by a specific loss of PCs. In addition, such a model would provide insight into the regenerative capacity of the cerebellum to replace lost PCs, for which genetic modifiers or regeneration-promoting compounds could be tested. We, therefore, established a transgenic zebrafish line designed PC-ATTAC™ (PC-specific Apoptosis Through Targeted Activation of Caspase 8 induced by Tamoxifen) whose PCs can be specifically and acutely ablated using Tamoxifen-inducible Caspase 8 activity. Carriers of the PC-ATTAC™ strain express two transgenes specifically in PCs: the fluorescent protein TagRFP as a reporter together with an initiation caspase, Caspase8, fused to a mutated Tamoxifen-interacting estrogen receptor ligand-binding domain (Casp8-ER^T2^) [152], both of which are linked by a self-cleavaging T2A peptide. In these animals, the red fluorescently-labeled PCs can be ablated by induced apoptosis mediated by the dimerization and activation of Casp8-ER^T2^ upon Tamoxifen treatment [81]. This model allowed for eliminating the majority of PCs (90%) when 4dpf old zebrafish PC-ATTAC™ larvae were exposed to Tamoxifen for 16 h. The induced apoptosis further induced accumulation of microglia engulfing and digesting ablated red fluorescent PC debris in the cerebellum. GCs and ECs, and their axonal and/or dendritic structures did not display any signs of cell death based on their morphology. This suggests that in this model GCs and ECs as afferent and efferent neurons of PCs can be studied with respect to their plasticity in response to a sudden PC loss and their contribution to a potential regeneration of PCs as described below. In addition, this model allowed for investigating the role of PCs in cerebellum-controlled behavior. PC ablated PC-ATTAC™ fish during larval stages displayed alterations in motor behavior such as an impaired optokinetic response, an enhanced swim speed, and increased thigmotaxis suggestive for increased anxiety [21]. In contrast, PC-ablated adult PC-ATTAC™ zebrafish did not overtly alter their locomotive behavior, whereas a compromised exploratory behavior could be observed [21], which is consistent with the behavioral impairment observed in SCA1 fish [23]. Noteworthy, another drug-inducible PC ablation model achieved by bacterial nitroreductase expression in PCs, resulted in a learning deficit of active avoidance in fear-conditioned adult zebrafish, evident in a slight reduction in their free-swimming speed [153]. These results underline again the striking role of PCs in regulating socio-emotional behaviors especially of adult zebrafish.

## Regeneration in the zebrafish cerebellum

**Fig. 4:**
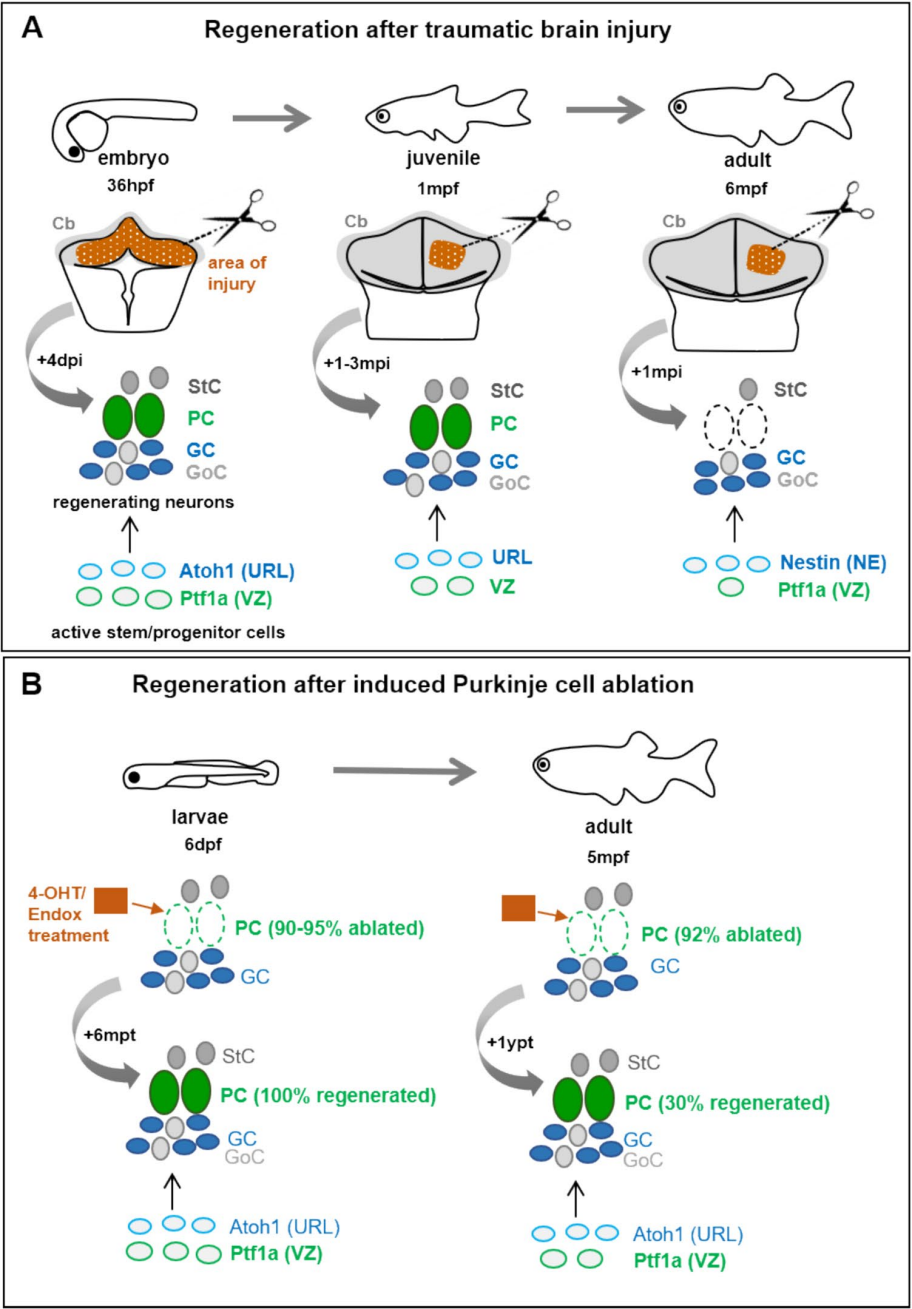
Schematic drawing of the cerebellar regeneration in zebrafish throughout development after acute physical injury into the differentiating (embryos) and mature (juveniles and adults) cerebellum (**A**), and regeneration of PCs after specific induced cell ablation in larvae and adult cerebellum (**B**). The area of the surgical lesion (highlighted in brown pattern), and the different cell types that appear to regenerate afterwards, together with their respective progenitors are illustrated. Information based on [19–21]. Abbreviations: Cb cerebellum, GC granule cells, GoC Golgi cells, hpf hours post-fertilization, mpf months post-fertilization, dpi days post-injury, mpi months post-injury, NE neuroepithelial-like cells, PC Purkinje cells, StC stellate cells, URL upper rhombic lip, VZ ventricular zone

Neurodegeneration leading to a loss of neurons could be counteracted by the regeneration of lost neuronal cells. Since zebrafish have a pronounced regenerative capacity of neuronal structures [154, 155], neurodegeneration models could not only serve to study the etiology of these diseases, but also to investigate regenerative responses to progressive neurodegeneration. The cerebellum of this bony fish holds an extraordinary regenerative capability. This is different from mammalians, in which the cerebellum is able to recover lost functions largely by plasticity, which has been termed the cerebellar reserve [156]. Various studies based on acute physical injuries in the cerebellum, from young embryos until adulthood, showed an age dependent potential for the healing and restoration of cerebellar tissue [19, 20]. The younger the more plastic its recovery is, but this ability appears more restricted to specific cell types with aging [20]. On the other hand, recently a new regeneration study based on specific induced ablation revealed that the main processing neurons of the cerebellum, the PCs, are capable of regenerating during the entire life (Fig. 4) [21].

### Regeneration in the developing and mature zebrafish cerebellum

#### after traumatic brain injury

At early developmental stages, it is possible to surgically remove the entire cerebellum and to recover the different cell types, such as upper rhombic lip-derived neurons and PCs [19]. The surgical removal of the developing cerebellum was performed at 36 hpf, followed by the reconnection of rhombic lips at the dorsal midline already at 72hpf, based on the expression of the early rhombic lip marker *zic1* in the dorso-anterior hindbrain. From time-lapse analysis, the ipsilateral dorso-lateral regions of the remaining hindbrain after ablation (and not from the most anterior *fgf8*-expressing isthmic band), were identified as the origin of the newly regenerating cells of the cerebellum. Later, at 6dpf the cerebellum recovered its regular structure, and mature PCs and granule cells were present (Fig. 4A). Furthermore, the differentiating cerebellum not only appeared able to recover at cellular level, but also functional recovery was obtained, since after a few days the swimming behavior of larvae appeared normal, showing control over body posture. Expression studies further disclosed that a repatterning process of the anterior hindbrain leads to the reestablishment of a developing cerebellar primordium at early stages [19]. However, this technical procedure of manually ablating the cerebellum is no longer possible at later developmental stages, due to side effects that such a severe injury might cause. In older fishes (juveniles and adults) only local damage by surgical injury or by blunt force can be applied [20, 157].

In juveniles (one month old zebrafish), the different neuronal populations endure their capacity to be restored after a stab lesion. In addition to the recovery of the granule cell layer, new PCs were present between 1 and 3 months post-injury (Fig. 4A). As putative precursors of regenerating PCs, a number of new proliferating *ptf1a +* cells (the natural source of cerebellar GABAergic neurons) were detected, even though these cell progenitors were reported to be significantly reduced in number in one month old fish [20].

Once the zebrafish reaches sexual maturity, the regeneration of adult brain structures is generally assisted by a teamwork of stem and progenitor cells from constitutive and reactive neurogenesis, which generate new neurons by both homeostatic mechanisms, and induced activation of wound healing after a damage, respectively.

Regarding the homeostatic production of neurons in the adult zebrafish cerebellum, the main contribution of new neurons occurs to the granule cell population. Proliferating granule cell precursors were detected as *nestin* + and *math1* + by [158], or *atoh1 +* by [159]. In addition, an increase of at least a few new PCs was observed after tracking cells for a period of 5 days, as well as some FABP7 + expressing, proliferating Bergman glia progenitors [159]. However, in other studies about adult neurogenesis, neither new PCs, nor proliferating radial glia were detected [158, 160]. A possible reason for these discrepancies might be due to the very slow production of these cell populations in adults.

Interestingly, similar to homeostatic conditions, after acute unilateral injury in the adult cerebellar cortex (affecting all cell types), mostly granule cells were restored [20]. BrdU pulse chase experiments demonstrated that most of newly regenerating cells after the induced lesion were granule cells. Only a few new GABAergic cells were detected, likely interneurons (Golgi and stellate cells), since none of them expressed PC markers (Fig. 4A). These findings suggest that the renewal of GABAergic cerebellar neurons in adult zebrafish becomes very restricted. The amount of proliferating stem and progenitor cells appeared consistent with the observations of the distribution of new mature cells: a significant and prolonged increase of proliferation of neuroepithelial-like *nestin +* cells (granule cell precursors), but only a small increase of *ptf1a +* proliferating cells (GABAergic cell precursors), and none of the new neurons appeared to originate from radial glial cells. Thus, different from other brain areas, the radial glia seems to play a minor role in cerebellar neuron regeneration [20]. Different types of traumatic brain injury by blunt force, produced consistent results in the specific increase of granule cell precursors [157]. Increase of *nestin +* cells during cerebellar regeneration has also been observed in juveniles of masu salmon [161].

Local injuries in the adult zebrafish brain triggers acute inflammation with intense microglia infiltration, and this inflammatory state seems to trigger a neuroregenerative response [162]. Such inflammation appears to influence neuroregeneration in the zebrafish cerebellum as well. Indeed, L-plastin-expressing microglia in the injured cerebellum are increased in number and activity for about one month after the injury, before returning to homeostatic levels [20].

Regeneration of local injuries in the cerebellum successfully restores its function since swimming defects observable several days after cerebellar injury had disappeared within one month after the injury [20].

Several studies on adult cerebellum regeneration by means of stab lesions, were also carried out in other species of teleost fish (*Apteronotus leptorhyncus*, or brown ghost knifefish). After an acute injury, the regenerative process follows a series of steps: (i) apoptotic response (a clean cell death for removing the damaged tissue and preventing scar formation), (ii) removal of dead cells by microglia cells (increase of lectin positive immune cells), and (iii) formation of new neurons, together with an increase in the density of radial glial cells near the lesion site [163–165]. The majority of the new cells are produced between 1 and 10 days after the lesion, of which at least a portion corresponds to new granule cell neurons [166]. Radial glia cells play a role in the guidance path for the migration of the newly regenerating cells towards the lesion site [163, 164]. When the regeneration process is more advanced at one-month post-lesion, the number of lectin-positive microglia returns to homeostatic levels [164].

These studies of acute brain injury let us gain insight into the regeneration capacities of the zebrafish cerebellum which, unlike mammalians, is possible to recuperate even in adults. Notwithstanding, the conducted physical lesions might hold some drawbacks, since they affect all cell types in only a restricted area, and leaving cells behind in the damaged area that could imply compensatory mechanisms by plasticity.

#### after cell type specific inducible cell ablation

Genetic ablation methods represent an alternative method for regeneration studies, in which a single but entire neuronal population is eliminated without affecting other cell types and precluding compensatory mechanisms from remaining cells. Such an approach could better mimic the conditions of degenerative diseases, where only certain neuronal types are affected. For instance, such methods are represented by ablations mediated by nitroreductase activation via metronidazole treatment, or mediated by caspase activity activated by Tamoxifen treatment to specifically induce the apoptosis of PCs [21, 81, 153]. Another specific ablation applicable to the zebrafish cerebellum is the laser excision of granule cell axons [92, 167] or the two-photon laser-mediated ablation of cells that has been applied for example to neurons of the inferior olive [168, 169]. Likewise, combinations with additional methods would be beneficial for further molecular studies on neurogenesis and regeneration, that would be valuable for learning more about the particular cellular and molecular mechanisms behind the restoration of each neuronal cell type in the cerebellum. For instance: (a) the generation of inducible CRISPR/Cas9 mutants for specific cell types, like for the progenitors of GABAergic neurons expressing *ptf1a* [170]; (b) making use of ‘neurospheres’, which allow the isolation and culture of specific stem/progenitor cells from the adult cerebellum [171]; (c) or zebrafish primary cell culture methods for specific neuronal cell types and their composition [172–174].

A recent study using specific PC ablation mediated by caspase activity mentioned above, indeed revealed that after the death of PCs in zebrafish larvae, the entire cell population is able to regenerate [21]. This full replenishment confirmed an actual regeneration process, rather than a partial continuation of the PC developmental process. *ptf1a +* cell progenitors of the ventricular zone appeared to be the main source of the newly regenerating PCs, and these *ptf1a +* cells correspond with the natural PC cell precursor in physiological conditions [159]. The functional recovery of the cerebellum was also demonstrated by electrophysiological and behavior analysis. Moreover, repeated ablation of PCs was repeatedly recovered by PC regeneration [21]. Furthermore, when inducing PC ablation in the adult cerebellum, new PCs also reappeared, that different from previous hypothesis, provided evidence for a maintenance of the regenerative capacity of the main cerebellar processing neurons throughout the entire life [21]. Interestingly, differential response during both degeneration and regeneration periods, were displayed along the anterior-posterior axis of the adult corpus cerebelli, as well as for different PC subtypes, being more resistant to cell death and prone to faster regeneration in the most posterior cerebellar area; the latter, akin to what frequently happens in diverse degenerative processes in mammalian cerebellum as well [21]. This study gained insight into the regeneration mechanisms of a specific neuronal population in the cerebellum after ablation. Such approach might better mimic deficiencies in neurodegenerative diseases, and thus represent a key tool for further studies on possible therapeutic treatments dealing with progressive PC loss in diseases such as cerebellar ataxias.

### Molecular clues to cerebellar regeneration in zebrafish

The large regenerative capability of the cerebellum, together with the multiple techniques and genetic tools accessible in zebrafish, render this bony fish an optimal model organism for developing future neuro-regenerative therapeutic strategies for the cerebellum. Nonetheless, for such a purpose, improvements in understanding two key aspects are needed: (a) revealing further the molecular mechanisms of neuronal regeneration and (b) investigating the evolutionary conservation of these mechanisms during development and regeneration. To date, some molecular processes involved in the recovery after acute surgical injury affecting a number of all cerebellar cell types at different developmental stages have been reported.

At embryonic stages, in vivo studies revealed that FGF signal transduction plays an essential role in the reestablishment of a cerebellar primordium and the regeneration of a differentiating cerebellum. Inhibition of FGF-signaling after cerebellum ablation in 36 hpf embryos drastically impaired its recovery. Thus, FGF-mediated repression of *hoxa2* gene expression in rhombomere 1 seems necessary for the restoration of the ablated embryonic cerebellum by hindbrain repatterning [19].

In adults, numerous genes have been linked to general brain regeneration processes i.e., genes expressed in common neurogenic areas, including the cerebellum, for instance, Krüppel-like factors, expressed in the granular cell area of the corpus cerebelli and valvula [175], or the Sprouty-related protein-2 (Spred-2), which stimulates cell proliferation after brain injury [176]. In addition, FGF signaling was reported to promote the proliferating activity of adult stem cells in the cerebellum [158], although whether it also plays a crucial role in cerebellar regenerative processes is not known so far.

Moreover, a few approaches specifically addressing the regeneration of the adult cerebellum in zebrafish showed molecular hints in promoting regeneration. For example, expression of Cadherin2 in granule cells in the larval cerebellum regulates coherent and directional migration [177]. Stab lesions in the adult cerebellum induced an increase of Cadherin2 expression surrounding the lesion site, which decreased to normal levels 30 days after, when regeneration was mostly completed, suggesting that Cadherin-2 could repeat its developmental role during adult granule cell regeneration [178]. In response to such lesions, cerebellar cells also appeared to express lectin, that might be related to the increase in microglia numbers at the lesion site [178]. More recently, regeneration following a traumatic brain injury by blunt force lesion revealed an influence of Shh signal transduction being involved in the proliferation of granule cell precursors [157]. After traumatic brain injury (by mechanical lesion in the cerebellum), protein-protein-interaction (PPI) networks were explored by time-course microarray analysis using wound-healing cerebellar tissue, and further integration of these data with available information in databases served to predict PPI signaling pathways, key for cerebellar regeneration [179]. Among the interactions, genes related to inflammation, neurogenesis and angiogenesis were predicted to play important regenerative roles. Indeed, these processes are commonly inherent of brain regenerative processes. Significant pathways described were: chemokine, Phosphatidylinositide 3-kinases, and axon guidance signaling pathways together with their cross-talks through PI3K and PLXNA2 [179]. Also, an additional bioinformatic analysis using microarray expression data and PPI examination after acute injury, compared core protein interactions in the cerebellum with regeneration processes in other organs, revealing core pathways such as TGF-β signaling during the blastema-like formation process. Organ specific proteins were disclosed as well, with the prediction of Wnt signaling as candidate pathway to be involved in proliferation (by Wnt3) and differentiation (by Ppp2ca) of neural stem cells during the regeneration process of the adult zebrafish cerebellum [180].

A proteomic analysis in other teleost fish species (*Apteronotus leptorhyncus*) revealed a collection of proteins likely involved in the regeneration of the adult cerebellum as well. From analysis of damaged and regenerating cerebellar tissue, an increase of proteins entailed to cellular proliferation, survival, and axonal sprouting, e.g.: β-Actin, β-Tubulin, Keratine 10, Lamin B2, Myosin-Heavy Chain and the neuropeptide Somatostatin, among others, were found [165, 181]. More recently, increase and decrease of certain proteins were also detected, already shortly after inducing a cerebellar lesion. Some of the functions of those proteins include e.g., blood clotting, energy metabolism, electron transfer in oxidative reactions, etc [182]. In addition, in the cerebellum of masu salmon, hydrogen sulfide appeared to be actively involved in the regeneration process during the post-traumatic response [161].

## Conclusions

Despite the various studies and multiple findings achieved so far, a number of regeneration promoting candidate molecules have been identified, but their functional involvement in cerebellar regeneration remains to be studied and confirmed. Hence, the molecular mechanisms of regeneration in the zebrafish cerebellum are far from being understood, but offer promising fields of study. Further molecular in vivo studies in zebrafish and other model organisms will be needed to corroborate the signaling pathways involved in cerebellar regeneration, as well as for deciphering distinctive mechanisms participating in the restoration of each specific cell type. Eventually, gaining insight into the induction of cerebellar regenerative processes in zebrafish could benefit the design of future therapeutic treatments for traumatic injuries and degenerative diseases affecting the cerebellum in humans.

## Supplementary Information 

Below is the link to the electronic supplementary material.

Supplementary Material 1 (PDF 173 KB)

## Data Availability

Not applicable.
