# Supplementary material for: Genetic modeling of degenerative diseases and mechanisms of neuronal regeneration in the zebrafish cerebellum
Source: Cell Mol Life Sci. 2024 Dec 27;82(1):26. doi: 10.1007/s00018-024-05538-z (PMC11671678; doi:10.1007/s00018-024-05538-z)
Supplement: Supplementary file 1 — Supplementary Material 1 (PDF 173 KB) [file 18_2024_5538_MOESM1_ESM.pdf]

Table. 1 Zebrafish models for SCA

SCA type (#OMIM)	Transgenic method [Reference]	Pathogenic transgene	Neurological phenotypes	Behavioral phenotypes	Additional phenotypes
SCA1 (#164400)	Stable transgenic expression using PC specific bidirectional promoter *(E1b:8xcpce:E1b promoter)* [1]	Human *ATXN1[82Q]*	• Progressive PC degeneration starting at 6 weeks-old.	• Less exploratory behavior measured by a novel tank diving test, starting at late larval stage of 6 weeks old until adulthood (3 months old).	
SCA3 (#109150)	Transient and ubiquitous expression by mRNA injection [2]	Human *ATXN3[80Q]*	• p53 dependent apoptotic neurons in brain at 24hpf.		
	Stable transgenic expression regulated by pan-neuronal *elavl3 (HuC)* promoter using Gal4/UAS system [3]	Human *ATXN3[84Q]*	• PolyQ positive neuritic beading pattern in the medulla region at adulthood (12 months-old).<br>• EGFP- ATXN3[84Q] aggregate formation in motoneurons at 6dpf.	• Reduced locomotive activity in free swimming at 6dpf<br>• Reduced locomotive activity in free swimming throughout late larval stage until adulthood (1 month to 12 months old).	• Detergent-insoluble ATXN3[Q82] particles at 2dpf.<br>• Increased cleaved ATXN3[Q82] fragments by calpain in whole zebrafish protein lysate.<br>• Shorter lifespan by 45days compared to control fish expressing ATXN3[Q23].
	Stable transgenic expression using motoneuron specific Gal4/UAS system regulated by *mir218* enhancer-*gata2* promoter [3]	Human *ATXN3[84Q]*	• significantly shorter axon lengths of spinal motoneurons in 48hpf zebrafish.	• Reduced locomotive activity in free swimming at 6dpf.<br>• Reduced locomotive activity in free swimming behavior at 3 months old.	
SCA13 (#605259)	Transient and ubiquitous expression by RNA injection [4]	Human $KCNC3^{R420H}$	• Suppressed excitability of fast-spiking caudal primary (CaP) motor neurons during evoked firing at 48-72hpf.	• Decreased precision and amplitude of the startle response at 55-60hpf.	
	Transient transgenic expression using motoneuron specific promoter (3 x *mnx1* enhancer-*gata2 minimal promoter*) [5]	Zebrafish $kcnc3a^{R335H}$ (equivalent to human $KCNC3^{R420H}$)	• Excessive distal axon branching of CaP motoneurons at 48hpf.		
		Zebrafish $kcnc3a^{F363L}$ (equivalent to human $KCNC3^{F448L}$)	• CaP motoneurons abnormally extending long, proximal collaterals into inappropriate synaptic territory at 48hpf.		
		Zebrafish $kcnc3a^{R335H}$ (equivalent to human $KCNC3^{R420H}$)	• Progressive PC degeneration staring at young larval stages prior to 7dpf.	• Impaired pursuit-Saccade eye movements in optokinetic response at 6dpf.	
	Transient transgenic expression using PC specific bidirectional promoter (E1b:2/4 x *cpce*:E1b) [6]	Zebrafish $kcnc3a^{R335H}$ (equivalent to human $KCNC3^{R420H}$)	• Suppressed excitability of PCs.<br>• Normally developed and matured PCs during larval stages.		
	Transient transgenic expression using PC specific *aldoca1*promoter [7]	Zebrafish $kcnc3a^{R338H}$ (equivalent to human $KCNC3^{R423H}$)	• Hyperexcitability of PCs transiently at 4.5pdf, PCs becoming inactive in subsequent larval stages.<br>• Impaired PC processes elongation with altered dendritic branching and synaptogenesis, resulting in cell death during larval stages.		
SCA37 (#615945)	Transient and ubiquitous expression by RNA injection [8]	RNA synthesized from Human mutated *Dab1* intronic fragment containing $(ATTTC)_{58}$			• Enhanced lethality and developmental malformations at 24dpf.
SCA49 (#619806)	Transient and ubiquitous expression by RNA injection [9]	Human $SAMD9L^{S626L}$	• Both WT SAMD9L and S626L mutant are localized in the mitochondria in neurons, and peripheral nerves in the hindbrain and spinal cord of 6dpf zebrafish, respectively.	• Larvae at 5dpf, expressing wild-type SAMD9L exhibited increased locomotive activity and head turns in the light-dark swimming test compared to uninjected larvae.<br>• Larvae expressing the $SAMD9L^{S626L}$ did not show these behavioral alterations.	• Enhanced DROP1 protein expression (indicative of mitochondrial biogenesis) in total lysate prepared from larvae expressing the $SAMD9L^{S626L}$.

Table. 2 Other zebrafish models in which SCA causative genes have been manipulated

Manipulated gene (human mutant causing SCA, #OMIM)	Gene manipulation ［Reference］	Neurological phenotypes	Behavioral phenotypes	Additional phenotypes
Human *ATAXN2* encoding an RNA metabolism regulator (*ATXN2- CAG* repeat expansion causing SCA2, #183090)	Transient and ubiquitous expression of intermediate length of ATNX2-polyQ (ATXN2[30Q]) by injection of its RNA along with the MO against C9orf72 to moderately (50-60%) reduce its expression [10]	• Disrupted arborization and shortening of the motor neuron axons in 48hpf zebrafish expressing ATXN2[30Q] along with moderately reduced C9orf72 expression.	• Reduced touch-evoked response in 48hpf zebrafish expressing ATXN2[30Q], along with moderately reduced C9orf72 expression.	
Zebrafish *atxn3* encoding a deubiquitinating enzyme (*ATXN3- CAG* repeat expansion causing SCA3, #109150)	MO-knockdown of *atxn3* [11]	• Higher dose of the MO injection resulted in a small head and reduced eye caused by retinal disorganization during larval stages.<br>• Lower dose of the MO injection caused impairments in photoreceptors: the rod outer segment elongation of rod photoreceptor and cone opsin mislocalization.		
Zebrafish *cacna1aa, and cacna1ab* encoding α1A subunits of the P/Q type voltage-gated Ca^{2+}channel (Cav2.1) (*CACNA1A-CAG* repeat expansion causing SCA6, #183086)	MO knockdown of *cacna1aa* [12]	• Spontaneous epileptiform-like discharges recorded in the optic tectum at 4dpf zebrafish injected with lower dose of the MO.	• Reduced touch response at 4dpf larvae injected with higher dose of the MO.<br>• Reduced locomotor activity in 4dpf larvae injected with lower dose of the MO during light-dark swimming.	• early embryonic lethality, or severely deformed embryos with higher dose of MO injection.<br>• curved body axis, small heads, tiny eyes, pericardial edema, yolk sac malformations in 4dpf larvae injected with higher dose of MO.<br>• Slightly hyperpigmented, no inflated swim bladder in 4dpf larvae injected with lower dose of the MO.
	Homozygous and homozygous mutation of *cacna1b* from intercrosses of heterozygous *tm154* (*fakir*) mutants (Cacna1ab^{L356V}) generated in ENU mutagenesis project) [13]	• Loss of touch-evoked motoneuron activation and fictive swimming at 48hpf in homozygous mutants, while NMDA-induced fictive swimming remains normal, indicating dysregulation of sensory transmission rather than that of neuromuscular system.	• Unresponsiveness, or reduced responsiveness (likely in homozygous, or heterozygous mutants, respectively), in touch-evoked swimming behaviors at 48hpf.	
	Morpholino knockdown of *cacna1ab* [13]		• Unresponsiveness in touch-evoked swimming at 48hpf.	
	Homozygous mutation of *cacna1ab* from intercrosses of heterozygous *tb204a* mutants (Cacna1ab^{Y1662N}) generated in ENU mutagenesis project [14]	• Impaired neuromuscular transmission caused by decreased intracellular Ca^{2+} in presynaptic neuromuscular junctions in 72-96hpf larval fish.	• Unresponsiveness of in homozygous mutants and reduced responsiveness of homozygous mutants in touch-evoked swimming during larval stages.	
Zebrafish *ataxin7* encoding a subunit of the STAGA transcription coactivator-HAT complex (human *ATAXIN7-CAG* repeat expansion causing SCA7, #164500)	Morpholino knockdown of zebrafish *atxn7* [15]	• Lower dose of the MO injection impaired differentiation of photoreceptors in retina, cerebellar PCs and granule neurons during larval stages.		• High dose of MO injection resulted in early embryonic lethality, or severely deformed embryos.
	MO knockdown of *atxn7* and Crispr/Cas9-mediated homozygous mutation of *atxn7* [16]	• Elevated Hedgehog signaling in the forebrain, altering proximo-distal patterning of the optic vesicle during early eye development, resulting in coloboma.<br>• Impaired axon pathfinding of retinal ganglion cells and optic nerve bundling at 5dpf.<br>• Incomplete formation of their outer segments along with reduced expression of *crx* transcription factor at 5dpf.		
Zebrafish *prkcg* encoding protein kinase C gamma (missense mutations in human *PRKCG* causing SCA14, #605361)	MO knockdown of zebrafish *prkcg* [17]	• The increases in AMPAmEPSCs (AMPA receptor miniature excitatory postsynaptic currents) amplitude, induced by Phorbol 12-myristate 13-acetate (PMA) and/or 5 mM K^+, was completely inhibited in Mauthner cells in the MO-injected larvae at 48hpf.	• Reduced responsiveness in touch-evoked swimming behavior at 48hpf when the chorion was removed manually.<br>• Unresponsiveness to sound or light stimulation.	• Failure of hatching, followed by death in subsequent several days.
Zebrafish *tpb* encoding TATA binding protein (tbp) (human *TBP-CAG/CAA* repeat expansion causing SCA17, #607136)	MO knockdown of zebrafish *tbp* [18]			• Epibolic movements during gastrulation stages were blocked, resulting in developmental arrest.<br>• Abolished expression of early patterning genes (*spadetail*, *goosecoid*, *even-skipped1*, *sonic hedgehog* and T-box6) at late blastula (at 4.5hpf), or early gastrula (at 6hpf) stage.

Table.3 Zebrafish models for neurodevelopmental disorders affecting cerebellum

Target disease (#OMIM)	Gene manipulation ［Reference］	Neurological phenotypes	Behavioral phenotypes	Additional phenotypes
Down Syndrome (#190685)	Stable transgenic expression of human Dyrk1A using PC specific bidirectional promoter (*E1b:4xcpce:E1b* promoter) [19]	• Condensed cerebellar hemispheres with more densely organized PCs, less complexity of PC dendrites at 4-7dpf.<br>• Disorganized PC layer with significantly reduced synaptic contacts in adult zebrafish.	• Reduced locomotive activity in free swimming behavior in 5dpf larvae<br>• Less exploratory behavior (novel tank diving test) in adult zebrafish older than 16 months.	
Ataxia-telangiectasia syndrome (#208900)	Morpholino knockdown of *ataxia–telangiectasia mutated* (*atm*) [20]			• Upon ionizing radiation-induced DNA damage, enhanced lethality and developmental defects (no pigmentation, lack of yolk extension, and extreme ventral body curvature) were observed in 6dpf zebrafish.
	CRISPR/Cas9-mediated homozygous mutation of *atm* [21]	• Enhanced neuroinflammation, gliosis, and oxidative stress in cerebellum and hindbrain.	• Reduced locomotive behavior in free swimming at 6 months old.	• A biased sex ratio (all are male) of *atm* homozygote mutant and infertility.<br>• Most fish died of a systemic infection before reaching 12 months of age, or developed malignant tumors.<br>• Increased tumorigenesis in kidney, splenomegaly at 12 months old<br>• A sign of myeloid leukemia: decreased the lymphocyte population as well as an increase in monocytes and precursor cells in kidney at 12 months old.<br>• Eye tumors causing exophthalmos at 12 months old.<br>• Myeloid and lymphoid cell lineages are impaired starting at 24hpf.
Autosomal recessive spastic ataxia of Charlevoix-Saguenay (ARSACS) (#270550)	CRISPR/Cas9-mediated homozygous mutation of *sacs* [22]	• Reduced PC area at 120hpf.<br>• Enhanced Ca^{2+} dynamics in PCs at 120hpf.	• Decreased tail flick frequency at 30hpf.<br>• Reduced locomotive behavior in dark-light swimming at 120hpf.<br>• Reduced locomotive behavior in free swimming behavior at 6 months old adult.	
Autosomal recessive SCA type 20 (SCAR20) (#616354)	Morpholino knockdown of zebrafish *sorting nexin 14* (*snx14*) [23]	• Reduced optic tectum and eye widths at 48hpf.<br>• Reduced progenitors of GABAergic cerebellar neurons at 36hpf.<br>• Reduced PC area in larval zebrafish.<br>• Increased number of activated caspase3 positive cells in brain at 36dpf.		
Pontocerebellar hypoplasia type 1B (PCH1B) (#614678)	Morpholino knockdown of *exosome component 3* (*exosc3*) [24]	• Accumulation of autophagic structures in neurons between optic lobes at 48hpf.<br>• Reduced brain size, especially in hindbrain region.<br>• Decreased expression of atoh1a (marker for dorsal hindbrain progenitors) in the upper and lower rhombic lip at 1dpf.	• Poor motility at embryonic stages	• Short and curved spines<br>• Enhanced mortality at 3dpf
Pontocerebellar hypoplasia type 1 (PCH1E) (#619303)	Morpholino knockdown of *slc25a46* [25]	• Decreased expression of pvalb7 (a marker for differentiated PCs) at 3dpf.	• Reduced locomotive behavior at 4dpf	• Severe curly-tail morphology in morphant embryos.
	Morpholino knockdown of *slc25a46* [26]	• Impaired RGC axon and dendrite formation in the tectum at 72hpf.<br>• Impaired RGC dendrite formation in the tectum at 96hpf.<br>• Brain maldevelopment (abnormal indentation at the midbrain-hindbrain junction, and abnormal gap between the optic tectum) in morphant embryos.		
Pontocerebellar hypoplasia type 2A/4/5(PCH2A/4/5) (#277470, #225753, #610204)	Morpholino knockdown of *tRNA-splicing endonuclease subunit 54* (*tsen54*) [27]	• Loss of spinal motor neurons in morphant embryos.<br>• Abnormally long and immotile mitochondria in cultured neurons prepared from 2dpf morphant embryos.		
	Homozygous premature stop-codon mutation from crosses between heterozygous *tens54*^{R228X} zebrafish generated in ENU mutagenesis project [27]			• Early lethality, dying by 9dpf

Target disease (#OMIM)	Genetic manipulation［Reference］	Neurological phenotypes	Behavioral phenotypes	Additional phenotypes
Pontocerebellar hypoplasia type 4 (PCH6) (#611523)	Morpholino knockdown of mitochondrial arginyl-tRNA synthetase (*rars2*) [27]	• Brain hypoplasia and loss of structural definition in the midbrain-hindbrain boundary at 24hpf.<br>• Increased cell death at 24hpf.		
Pontocerebellar hypoplasia type 7 (PCH7) (#614969)	Morpholino knockdown of *toe1* [28]	• Reduced head and eye sizes by 48hpf.<br>• Hypoplasia in the midbrain, cerebellum, and hindbrain showing apoptotic cells at 48hpf	• reduced swimming behavior at 96hpf when stimulated by light and sound	• Thin and curly tail at 48hpf
	Morpholino knockdown of *tbc1d23* [29]	• Hypoplasia in the forebrain, brainstem, and cerebellum at 48hpf.		• Curly tail at 48hpf
Pontocerebellar hypoplasia type 11 (PCH11) (#617695)	Morpholino knockdown of *tbc1d23* [30]	• Enlarged 4th ventricle at 48hpf.<br>• Loss of HuC positive panneuronal cells at 48hpf.<br>• Loss of GFAP positive glia cells at 48hpf.<br>• Abnormally branched axons of CaP motor neurons at 48hpf.		• Curly tail at 48hpf
Pontocerebellar hypoplasia type 17 (PCH17) (#619909)	Homozygous premature stop-codon mutation of *prdm13* from a cross between heterozygous *prdm13*^{sa16464} zebrafish, ENU mutagenesis project [31]	• Reduced PCs, whereas Eurydendroid cells were not affected at 7dpf<br>• Complete loss of neurons in inferior olivary nucleus at7dpf<br>• Enhanced larval lethality at 9dpf<br>• Reduced head size, and cerebellar hypoplasia at 3dpf		• Although the overall morphology appeared roughly normal during larval stages, an abnormal body curvature and lower jaw morphology were observed at 7dpf
Intellectual developmental disorder with microcephaly and pontine and cerebellar hypoplasia (MICPCH) (#300749)	Morpholino knockdown of *caska* and *caskb* [32]	• 10% , or 13% reduction in head size in *caska*, or *caskb* morphants at 3dpf, respectively.<br>• Cerebellar axons visualized with anti-acetylated tubulin staining were severely affected in both *caska* and *caskb* morphants at 3dpf.		• Short and curved spines<br>• Enhanced mortality rate at 3dpf
Joubert Syndrome (40 subtypes registered in OMIM)	The list of the zebrafish morphants and mutant models for Joubert Syndrome, and their phenotypes are provided in [33]	.		

References

1. Elsaey MA, Namikawa K, Köster RW (2021) Genetic Modeling of the Neurodegenerative Disease Spinocerebellar Ataxia Type 1 in Zebrafish. Int. J. Mol. Sci. 22

2. Liu H, Li X, Ning G, et al (2016) The Machado–Joseph Disease Deubiquitinase Ataxin-3 Regulates the Stability and Apoptotic Function of p53. PLoS Biol 14(11):e2000733. https://doi.org/10.1371/journal.pbio.2000733

3. Watchon M, Yuan KC, Mackovski N, et al (2017) Calpain Inhibition Is Protective in Machado–Joseph Disease Zebrafish Due to Induction of Autophagy. J Neurosci 37:7782 LP – 7794. https://doi.org/10.1523/JNEUROSCI.1142-17.2017

4. Issa FA, Mazzochi C, Mock AF, Papazian DM (2011) Spinocerebellar Ataxia Type 13 Mutant Potassium Channel Alters Neuronal Excitability and Causes Locomotor Deficits in Zebrafish. J Neurosci 31:6831 LP – 6841. https://doi.org/10.1523/JNEUROSCI.6572-10.2011

5. Issa FA, Mock AF, Sagasti A, Papazian DM (2012) Spinocerebellar ataxia type 13 mutation that is associated with disease onset in infancy disrupts axonal pathfinding during neuronal development. Dis Model Mech 5:921–929. https://doi.org/10.1242/dmm.010157

6. Namikawa K, Dorigo A, Zagrebelsky M, et al (2019) Modeling Neurodegenerative Spinocerebellar Ataxia Type 13 in Zebrafish Using a Purkinje Neuron Specific Tunable Coexpression System. J Neurosci 39:3948–3969. https://doi.org/10.1523/JNEUROSCI.1862-18.2019

7. Hsieh J-Y, Ulrich BN, Issa FA, et al (2020) Infant and adult SCA13 mutations differentially affect Purkinje cell excitability, maturation, and viability in vivo. Elife 9:e57358. https://doi.org/10.7554/eLife.57358

8. Seixas AI, Loureiro JR, Costa C, et al (2017) A Pentanucleotide ATTTC Repeat Insertion in the Non-coding Region of *DAB1*, Mapping to SCA37, Causes Spinocerebellar Ataxia. Am J Hum Genet 101:87–103. https://doi.org/10.1016/j.ajhg.2017.06.007

9. Corral-Juan M, Casquero P, Giraldo-Restrepo N, et al (2022) New spinocerebellar ataxia subtype caused by SAMD9L mutation triggering mitochondrial dysregulation (SCA49). Brain Commun 4:fcac030. https://doi.org/10.1093/braincomms/fcac030

10. Sellier C, Campanari M, Julie Corbier C, et al (2016) Loss of C9ORF72 impairs autophagy and synergizes with polyQ Ataxin-2 to induce motor neuron dysfunction and cell death. EMBO J 35:1276–1297. https://doi.org/https://doi.org/10.15252/embj.201593350

11. Toulis V, García-Monclús S, de la Peña-Ramírez C, et al (2020) The Deubiquitinating Enzyme Ataxin-3 Regulates Ciliogenesis and Phagocytosis in the Retina. Cell Rep 33:. https://doi.org/10.1016/j.celrep.2020.108360

12. Gawel K, Turski WA, van der Ent W, et al (2020) Phenotypic Characterization of Larval Zebrafish (Danio rerio) with Partial Knockdown of the cacna1a Gene. Mol Neurobiol 57:1904–1916. https://doi.org/10.1007/s12035-019-01860-x

13. Low SE, Woods IG, Lachance M, et al (2012) Touch responsiveness in zebrafish requires voltage-gated calcium channel 2.1b. J Neurophysiol 108:148–159. https://doi.org/10.1152/jn.00839.2011

14. Wen H, Linhoff MW, Hubbard JM, et al (2013) Zebrafish Calls for Reinterpretation for the Roles of P/Q Calcium Channels in Neuromuscular Transmission. J Neurosci 33:7384 LP – 7392. https://doi.org/10.1523/JNEUROSCI.5839-12.2013

15. Yanicostas C, Barbieri E, Hibi M, et al (2012) Requirement for Zebrafish Ataxin-7 in Differentiation of Photoreceptors and Cerebellar Neurons. PLoS One 7:e50705. https://doi.org/10.1371/journal.pone.0050705

16. Carrillo-Rosas S, Weber C, Fievet L, et al (2019) Loss of zebrafish Ataxin-7, a SAGA subunit responsible for SCA7 retinopathy, causes ocular coloboma and malformation of photoreceptors. Hum Mol Genet 28:912–927. https://doi.org/10.1093/hmg/ddy401

17. Patten SA, Roy B, Cunningham ME, et al (2010) Protein kinase Cγ is a signaling molecule required for the developmental speeding of α-amino-3-hydroxyl-5-methyl-4-isoxazole-propionate receptor kinetics. Eur J Neurosci 31:1561–1573. https://doi.org/https://doi.org/10.1111/j.1460-9568.2010.07216.x

18. Müller F, Lakatos L, Dantonel J-C, et al (2001) TBP is not universally required for zygotic RNA polymerase II transcription in zebrafish. Curr Biol 11:282–287. https://doi.org/10.1016/S0960-9822(01)00076-8

19. Buchberger A, Schepergerdes L, Flaßhoff M, et al (2021) A novel inhibitor rescues cerebellar defects in a zebrafish model of Down syndrome-associated kinase Dyrk1A overexpression. J Biol Chem 297:100853. https://doi.org/10.1016/j.jbc.2021.100853

20. Imamura S, Kishi S (2005) Molecular cloning and functional characterization of zebrafish ATM. Int J Biochem Cell Biol 37:1105–1116. https://doi.org/https://doi.org/10.1016/j.biocel.2004.10.015

21. Chen K, Wang P, Chen J, et al (2022) Loss of atm in Zebrafish as a Model of Ataxia–Telangiectasia Syndrome. Biomedicines 10(2). 392. https://doi.org/10.3390/biomedicines10020392

22. Naef V, Marchese M, Ogi A, et al (2021) Efficient Neuroprotective Rescue of Sacsin-Related Disease Phenotypes in Zebrafish. Int. J. Mol. Sci. 22(16) 8401. https://doi.org/10.3390/ijms22168401

23. Akizu N, Cantagrel V, Zaki MS, et al (2015) Biallelic mutations in SNX14 cause a syndromic form of cerebellar atrophy and lysosome-autophagosome dysfunction. Nat Genet 47:528–534. https://doi.org/10.1038/ng.3256

24. Wan J, Yourshaw M, Mamsa H, et al (2012) Mutations in the RNA exosome component gene EXOSC3 cause pontocerebellar hypoplasia and spinal motor neuron degeneration. Nat Genet 44:704–708. https://doi.org/10.1038/ng.2254

25. Abrams AJ, Hufnagel RB, Rebelo A, et al (2015) Mutations in SLC25A46, encoding a UGO1-like protein, cause an optic atrophy spectrum disorder. Nat Genet 47:926–932. https://doi.org/10.1038/ng.3354

26. Wan J, Steffen J, Yourshaw M, et al (2016) Loss of function of SLC25A46 causes lethal congenital pontocerebellar hypoplasia. Brain 139:2877–2890. https://doi.org/10.1093/brain/aww212

27. Kasher PR, Namavar Y, van Tijn P, et al (2011) Impairment of the tRNA-splicing endonuclease subunit 54 (tsen54) gene causes neurological abnormalities and larval death in zebrafish models of pontocerebellar hypoplasia. Hum Mol Genet 20:1574–1584. https://doi.org/10.1093/hmg/ddr034

28. Lardelli RM, Schaffer AE, Eggens VRC, et al (2017) Biallelic mutations in the 3′ exonuclease TOE1 cause pontocerebellar hypoplasia and uncover a role in snRNA processing. Nat Genet 49:457–464. https://doi.org/10.1038/ng.3762

29. Marin-Valencia I, Gerondopoulos A, Zaki MS, et al (2017) Homozygous Mutations in TBC1D23 Lead to a Non-degenerative Form of Pontocerebellar Hypoplasia. Am J Hum Genet 101:441–450. https://doi.org/10.1016/j.ajhg.2017.07.015

30. Huang W, Liu Z, Yang F, et al (2019) Structural and functional studies of TBC1D23 C-terminal domain provide a link between endosomal trafficking and PCH. Proc Natl Acad Sci 116:22598–22608. https://doi.org/10.1073/pnas.1909316116

31. Coolen M, Altin N, Rajamani K, et al (2022) Recessive PRDM13 mutations cause fatal perinatal brainstem dysfunction with cerebellar hypoplasia and disrupt Purkinje cell differentiation. Am J Hum Genet 109:909–927. https://doi.org/10.1016/j.ajhg.2022.03.010

32. Cristofoli F, Devriendt K, Davis EE, et al (2018) Novel CASK mutations in cases with syndromic microcephaly. Hum Mutat 39:993–1001. https://doi.org/https://doi.org/10.1002/humu.23536

33. Rusterholz TDS, Hofmann C, Bachmann-Gagescu R (2022) Insights Gained From Zebrafish Models for the Ciliopathy Joubert Syndrome. Front Genet 13: https://doi.org/10.3389/fgene.2022.939527
